# Effects of the repression of *GIGANTEA* gene *StGI.04* on the potato leaf transcriptome and the anthocyanin content of tuber skin

**DOI:** 10.1186/s12870-022-03636-3

**Published:** 2022-05-20

**Authors:** Khongorzul Odgerel, Jeny Jose, Flóra Karsai-Rektenwald, Gitta Ficzek, Gergely Simon, György Végvári, Zsófia Bánfalvi

**Affiliations:** 1 Genetics and Biotechnology Institute, Hungarian University of Agriculture and Life Sciences, Szent-Györgyi A. u. 4, Gödöllő, H-2100 Hungary; 2 Centre for Agricultural Research, Eötvös Loránd Research Network, Brunszvik u. 2, Martonvásár, H-2462 Hungary; 3 Department of Fruit Growing, Institute of Horticulture, Hungarian University of Agriculture and Life Sciences, Villányi út 29-43, Budapest, H-1118 Hungary; 4 Institute of Viticulture and Oenology, Faculty of Natural Sciences, Eszterházy Károly Catholic University, Eszterházy tér 1, Eger, H-3300 Hungary

**Keywords:** Anthocyanins, Antisense repression, GIGANTEA, *Solanum tuberosum*, Transcriptome, Tuberisation, Tuber skin colour

## Abstract

**Background:**

GIGANTEA (GI) is a plant-specific, circadian clock-regulated, nuclear protein with pleiotropic functions found in many plant species. This protein is involved in flowering, circadian clock control, chloroplast biogenesis, carbohydrate metabolism, stress responses, and volatile compound synthesis. In potato (*Solanum tuberosum* L.), its only role appears to be tuber initiation; however, based on findings in other plant species, we hypothesised that the function of GI in potatoes is not restricted only to tuberisation.

**Results:**

To test this hypothesis, the expression of a *GI* gene in the commercial potato cultivar ‘Désirée’ was repressed, and the effects of repression at morphological and transcriptome level were investigated. Previously, two copies of *GI* genes in potato were found. A construct to reduce the mRNA levels of one of these genes (*StGI.04*) was assembled, and the effects of antisense repression were studied in greenhouse-grown plants. The highest level of repression reached around 50%. However, this level did not influence tuber formation and yield but did cause a reduction in tuber colour. Using high-performance liquid chromatography (HPLC), significant reductions in cyanidin 3,5-di-O-glucoside and pelargonidin 3,5-di-O-glucoside contents of tuber peels were detected. Anthocyanins are synthesized through a branch of the phenylpropanoid pathway. The transcriptome analysis indicated down-regulation in the expression of *PHENYLALANINE AMMONIA LYASE* (*PAL)*, the *LEUCOANTHOCYANIDIN OXIDISING* enzyme gene *LDOX*, and the *MYB-RELATED PROTEIN Hv1* (*MYB-Hv1*)*,* a transcription factor coding gene, which is presumably involved in the regulation of flavonoid biosynthesis, in the leaves of a selected *StGI.04*-repressed line. Furthermore, alterations in expression of genes affecting the circadian clock, flowering, starch synthesis, and stress responses were detected in the leaves of the selected *StGI.04*-repressed line.

**Conclusions:**

We tested the effects of antisense repression of *StGI.04* expression in potatoes and found that as with GI in other plant species, it influences the expression of the key genes of the circadian clock, flowering, starch synthesis, and stress responses. Furthermore, we detected a novel function of a *GI* gene in influencing the anthocyanin synthesis and potato tuber skin colour.

**Supplementary Information:**

The online version contains supplementary material available at 10.1186/s12870-022-03636-3.

## Background

GIGANTEA (GI) is a plant-specific, circadian clock-regulated, nuclear protein with pleiotropic functions. The most extensively studied physiological process in which GI is involved is flowering. It has been shown in several plant species that GI regulates flowering time through the photoperiod-pathway. It is indicated by the *gi Arabidopsis* mutants that GI acts in the long-day (LD) flowering pathway because *gi* mutants flower late under LD conditions. GI forms a complex with the FLAVIN-BINDING KELCH-REPEAT F-Box 1 (FKF1) protein and up-regulates the expression of *CONSTANS* (*CO*) by degrading the *CO*-repressor, CYCLING DOF FACTOR 1 (CDF1). CO measures the duration of daytime and activates *FLOWERING LOCUS T* (*FT*), encoding the mobile peptide ‘florigen’, and *TWIN SYSTER OF FT* (*TSF*) under LD conditions (reviewed in [1]). Recently, it has been shown that the GI-FKF1-CDF1-CO module is employed even by mangoes in regulating its temperature dependent flowering [2].

Besides flowering time regulation, GI is involved in circadian clock control while the expression of *GI* itself is regulated by the circadian clock and peaks 8–10 hours after dawn. GI interacts with several clock proteins. It also functions in the process of light signalling. It appears to be a positive regulator of *PHYTOCHROME B* (*PHYB*) signalling. The *gi* mutant *Arabidopsis* seedlings possess long hypocotyls under blue light indicating that GI has a role also in the blue light signalling. Furthermore, GI functions in chloroplast biogenesis and chlorophyll accumulation; the *gi* mutants are characterised by increased chlorophyll level. GI has a direct connection with the sucrose metabolism. Increased starch content was observed in *Arabidopsis* and rice *gi* mutants. GI interacts with TREHALOSE-6-PHOSPHATE SYNTHASE 8 and this interaction may have a direct influence on the carbohydrate metabolism (reviewed in [3]).

Another large category of biological functions, in which GI is involved, is the responses of plants to environmental stresses. GI acts by conferring salt and freezing tolerance to *Arabidopsis* and *Brassica nigra*. In contrast, in *B. rapa* and poplar plants, down-regulation of *GI* leads to enhancement of salt tolerance. *GI* expression is induced in response to drought stress and in combination with miRNA172, causes suppression of *WRKY44*, a transcription factor participating in sugar metabolism. GI plays an inhibitory role during oxidative stress by causing down-regulation of the expression of *SUPEROXIDE DISMUTASE* (*SOD*) and *ASCORBATE PEROXIDASE* (*APX*) (reviewed in [4]). The latest results show that GI influences not only abiotic stress responses but is also involved in response to pathogen defence, and it confers susceptibility to plants during spot blotch attack by regulating the salicylic acid signalling pathway [5].

Brandoli et al. [6] reported a novel role of *GI* in *Petunia hybrida*. Flowers of the *GI* silenced lines emitted 20% less volatile compounds on a fresh weight basis over 24 h and showed changes in the scent profile. The relative abundance of the trans-cinnamic acid derivatives whose precursor is phenylalanine showed alterations, especially in the morning.

In potato (*Solanum tuberosum* L.), involvement of *GI* in initiation of tuberisation was demonstrated by Kloosterman et al. [7]. According to the proposed model based on studying the wild Andean landrace *S. tuberosum* Group Andigena, a strict short-day (SD) plant for tuberisation, GI influences tuber formation in conjunction with FKF1. As in *Arabidopsis*, the GI-FKF1 complex can bind CDF1 and target it for degradation by the proteasome. Since CDF1, in an indirect way, increases the transcription of *SELF-PRUNING 6A* (*SP6A*), a mobile tuberisation signal homologous to the ‘florigen’ FT, GI is an indirect repressor of tuberisation.

Recently, searching for the *A. thaliana GI* homologue we found two *GI* transcript variants in potato with 83.73% identity located on chromosomes 4 and 12 and designated them *StGI.04* and *StGI.12*. In silico characterisation and expression analysis of the two genes revealed that their regulation is partially different. While osmotic stress, cold stress, and heat lead to up-regulation of *StGI.04*, the same stresses produce down-regulation in the expression of *StGI.12*. ABA induces *StGI.12* but has no effect on *StGI.04* [8]. Besides tuberisation, no detailed characterisation of the *GI* genes has been reported thus far in potato; therefore, it was decided to study their function by antisense repression of gene expression starting with *StGI.04*. Based on the phenotype of the plants in addition to the effects of *StGI.04* repression on the potato leaf transcriptome, a novel role of GI in anthocyanin metabolism was demonstrated and similarities and differences in influencing gene expression in comparison with other plant species were highlighted.

## Results

### Selection of *StGI.04*-repressed lines

**Fig. 1:**
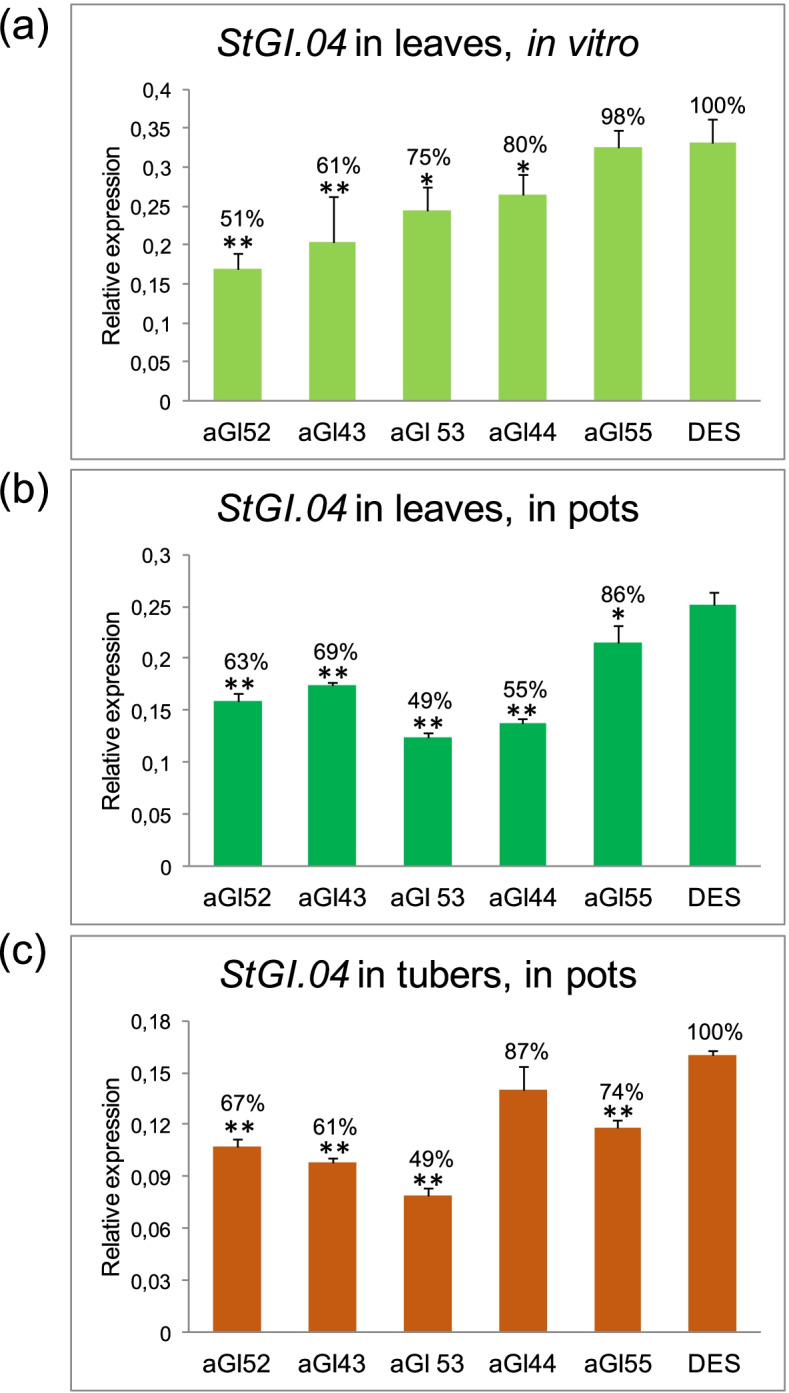
Level of *StGI.04* repression in aGI lines compared to the non-transformed control ‘Désirée’ (DES). RNA was isolated from (**a**) middle leaves of in vitro plants; 3 leaves/line harvested from 3 plants, (**b**) source leaves of 8-week-old plants grown in pots in a greenhouse; 9 leaf discs of 1 cm in diameter/line harvested from 9 plants, (**c**) mature tubers harvested at the end of the vegetation period; 3 sets/line, 3 tubers/set composed from the largest tubers of each line distributed into approximately equal groups. Y-axis shows mean relative expression values of *StGI.04* gene compared to the mean expression values of *ACTIN*. The means were calculated from three technical replicates in case of (**a**) and (**b**) and from 3 biological replicates in case of (**c**). The standard deviations are indicated by the error bars. Significant differences between the aGI lines and DES were detected by Student’s *t*-test and labelled by * (*p* < 0.05) and ** (*p* < 0.01)

A 250-bp fragment of *St.GI04* with the less, but still 71.2% identity with the corresponding fragment of *StGI.12* (Additional file 1: Fig. S1), was used for generation of *StGI.04*-repressed ‘Désirée’ (DES) lines (aGI lines). Leaves harvested from plants grown in vitro were tested for the level of repression using reverse-transcription polymerase chain reaction (RT-PCR). Twenty lines were found with lower level of *StGI.04* expression than the non-transformed control DES out of which five lines (aGI43, aGI44, aGI52, aGI53, aGI55) with different levels of reduction in *StGI.04* transcript level were selected for further studies. Expression of *StGI.04* was quantified in the selected five lines with reverse transcription-quantitative polymerase chain reaction (RT-qPCR). The highest repression, 51% of *StGI.04* mRNA detected in DES, was found in aGI52, while only a minimal repression, a 2% reduction, was found in aGI55 (Fig. 1a).

The five selected lines were propagated in vitro, transferred to pots, and grown further under greenhouse conditions in 12 parallel setups. Expression of *StGI.04* was re-tested in leaves (Fig. 1b). Four lines possessed significantly (*p* < 0.01) lower *StGI.04* expression than found in DES. As in leaves of in vitro plants, a smaller difference compared to DES was found in aGI55, but even this difference was significant at the *p* < 0.05 level. The *StGI.04* transcript level was a little bit higher, 63% versus 51%, in aGI52 leaves of greenhouse-grown plants versus leaves of in vitro-grown plants.

At the end of the vegetation period, the tubers were harvested, and the level of *StGI.04* expression was tested in tubers (Fig. 1c). In line with the lowest expression in leaves (Fig. 1b), the lowest expression was detected in tubers of the aGI43 plants (Fig. 1c). All lines, except aGI44, showed a significant (*p* < 0.01) level of *StGI.04* repression in tubers, including aGI52 with 67% of wild type *StGI.04* mRNA level.

### Specificity of repression in aGI52

The line aGI52 showed a significant (*p* < 0.01) and relatively stable level of reduction in *StGI.04* expression based on all three RT-qPCR analyses (Fig. 1). Therefore, this line was selected for further detailed studies.

**Fig. 2:**
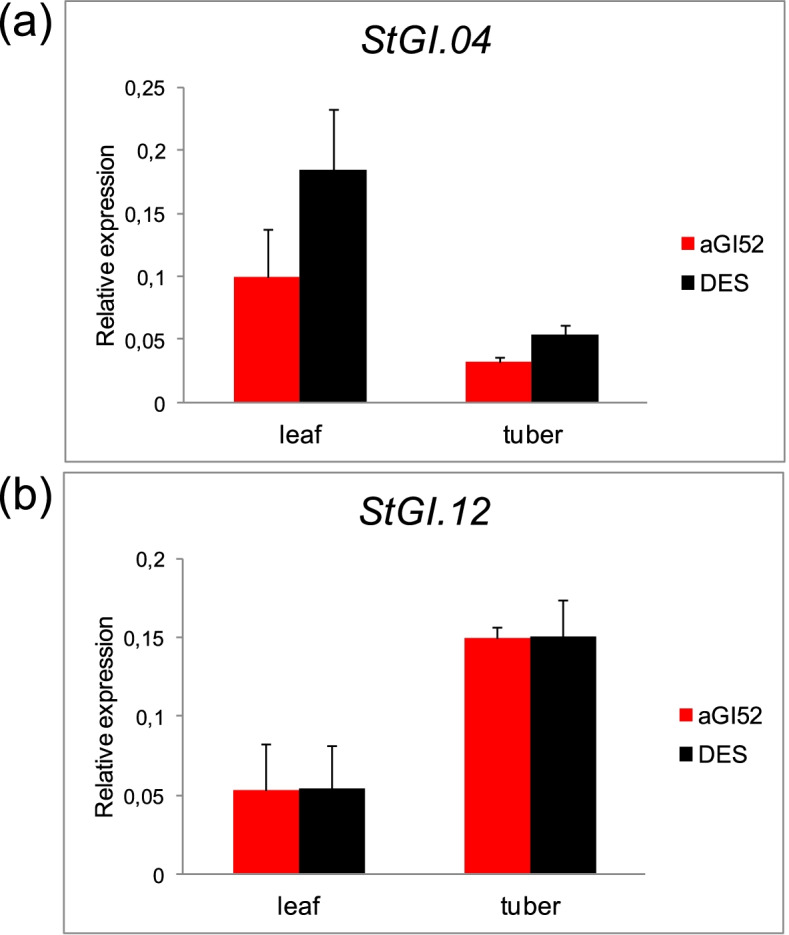
Specificity of *StGI.04* repression in aGI52. The RT-qPCR analysis was carried out using (**a**) the GI04spec and (**b**) the GI12spec primer pair. The leaf RNA was derived from 1-cm-diameter discs of 9 leaves of 9 plants divided into 3 groups. The tuber RNA was the same used in the RT-qPCR analysis presented in Fig. 1c. Y-axis shows mean relative expression values of *StGI* genes compared to the geometric mean expression values of *ACTIN* and *EF1α*. The standard deviations from three biological replicates are indicated by the error bars. DES, non-transformed control ‘Désirée’

Although the region of reduced sequence similarity between *StGI.04* and *StGI.12* was used for generation of aGI lines, the identity of the two regions was still substantial. Thus, the repression of *StGI.12* expression by the *StGI.04* fragment could not be excluded. To test this possibility, an *StGI.12*-specific primer pair [8] was used in parallel with the *StGI.04*-specific primer pair [8] in the RT-qPCR analysis of aGI52 and DES leaves and tubers. Figure 2 shows that the repression in aGI52 was *StGI.04*-specific and did not extend to *StGI.12*.

### Phenotypes and tuberisation of aGI plants

Development and morphology of aGI43, aGI44, aGI52, aGI53, and aGI55 plants grown in the greenhouse were visually followed and compared to DES. Plant heights were measured at 7 weeks after transferring them from in vitro into pots. Neither phenotypic changes nor height differences were observed. The earliness of tuberisation was tested also at 7 weeks after planting by counting the number of tubers after carefully tipping the plants out of the pots. Significant delay in tuber initiation was detected only in the line aGI44. After counting, the plants were replaced in the pots and grown until the end of the vegetation period when the tubers were harvested and measured for weight. No differences in tuber yield were obtained between the aGI lines and DES. The size distribution of aGI tubers was also similar to DES, peaking at 8 to 10 cm in diameter, except aGI44 and aGI55, both of which produced a larger number of small tubers than DES (Additional file 1: Fig. S2).

The molecular model of tuber formation is based on *S. andigena*, a strict SD plant for tuberisation [7]. Therefore, we wanted to test the effects of *StGI.04* repression not only under 12 h light/12 h dark (LD conditions) but also under SD conditions (8 h light/16 h dark). The aGI52 line was compared to DES in this experiment. Even under SD conditions, no difference in canopy phenotype or tuber yield was detected between aGI52 and DES (Additional file 1: Fig. S2).

### Anthocyanin content of tuber peels

**Fig. 3:**
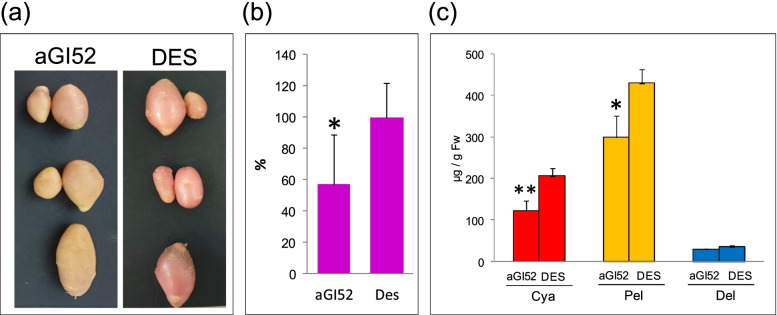
Anthocyanin content of aGI52 and DES tubers grown under SD conditions. **a** Morphology of mature tubers of three aGI52 and three non-transformed, control DES plants. **b** The relative anthocyanin content of tuber skins determined spectrophotometrically. **c** The anthocyanin content of tuber skins analysed using HPLC. Abbreviations: Cya, cyanidin 3,5-di-O-glucoside; Pel, pelargonidin 3,5-di-O-glucoside; Del, delphinidin 3,5-di-glycoside. Tuber skins were peeled from 3 sets of largest tubers per line; each set contained 3 tubers. The extraction was from equal amounts of skins. The standard deviations are indicated by the error bars. Significant differences between aGI52 and DES were detected by Student’s *t*-test and labelled by * (*p* < 0.05) and ** (*p* < 0.01). Fw, fresh weight

DES is a red-skinned potato. The skin colour of aGI tubers collected from greenhouse-grown plants was lighter than the skin colour of DES (Additional file 1: Fig. S3a) although, to different extents. The reduction in colour was the most pronounced in aGI52, aGI53 and aGI44. The difference in tuber skin colour was also obvious between aGI52 and DES grown under SD conditions (Fig. 3a). Since anthocyanins determine the skin colour [9], these compounds were extracted from tuber peels and their relative quantity measured. When compared with DES, a 52, 36 and 31% reduction in anthocyanin content was found in greenhouse-grown aGI53, aGI44 and aGI52 tuber peels, respectively (Additional file 1: Fig. S3b). A similar, 43% reduction was observed in the anthocyanin content of aGI52 tuber skins developed under SD conditions (Fig. 3b).

High-performance liquid chromatography (HPLC) was used to specify the anthocyanins extracted from tuber peels. Three anthocyanins, cyanidin 3,5-di-O-glucoside, pelargonidin 3,5-di-O-glucoside and delphinidin 3,5-di-glycoside were detected with pelargonidin 3,5-di-O-glycoside being present in the largest amount. A significant reduction in cyanidin 3,5-di-O-glucoside and pelargonidin 3,5-di-O-glycoside content of aGI52 peels compared to DES peels was demonstrated (Fig. 3c). An attempt was also made to detect malvinidin 3-galactoside; however, it was not present in a detectable amount.

### Transcriptome analysis of aGI52 leaves

To explore the effects of *StGI.04* repression on the global transcription profile, the same leaf RNA of greenhouse-grown aGI52 and DES plants tested for the specificity of repression (Fig. 2) was used for RNA-seq analysis in three biological replicates. Parameters presented in Additional files Table S1, Fig. S4 and S5 indicate that the analysis had a good quality.

**Fig. 4:**
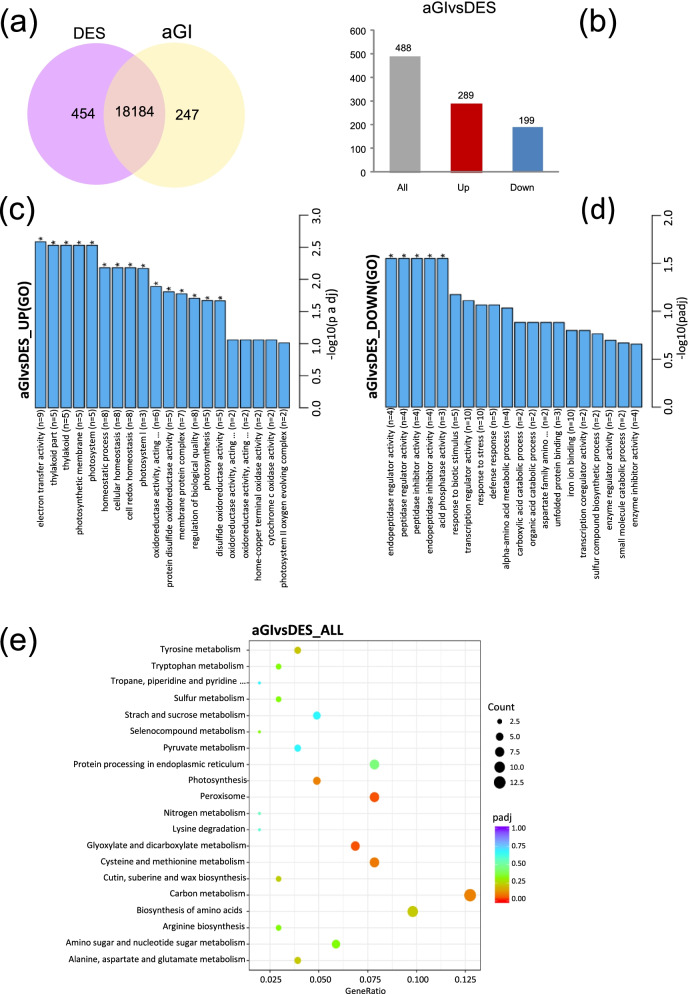
Identification of differentially expressed genes in aGI52 leaves compared to the non-transformed control ‘Désirée’ (DES) leaves and KEGG pathway analysis. **a** Venn diagram of differentially expressed genes. The overlap represents the genes expressed in common in aGI52 and DES. **b** Number of up-regulated and down-regulated genes. **c** Bar diagram showing the up-regulated GOs. **d** Bar diagram showing the down-regulated GOs. The number of DEGs is shown in parenthesis. The significantly enriched GO terms are marked by asterisks. **e** KEGG [10] enrichment scatter plot of DEGs

To assess the alterations in unigene expression we analysed the differentially expressed unigenes (|log2 (Fold Change)| > 1 and padj < 0.05) in the comparison of aGI52 and DES. We found 454 and 247 uniquely expressed genes in DES and aGI52, respectively (Fig. 4a). We obtained 488 differentially expressed genes (DEGs): (1) 289 were up- and (2) 199 were down-regulated in aGI52 (Fig. 4b and Additional file 1: Fig. S6). Gene ontology (GO) enrichment analysis revealed that mainly those genes were up-regulated, which are related to photosynthesis, while peptidase regulators and inhibitors were down-regulated in aGI52 (Fig. 4c, d and Additional file 1: Fig. S7). The Kyoto Encyclopedia of Genes and Genomes (KEGG) analysis showed that the glyoxylate and dicarboxylate metabolism, the carbon metabolism and the peroxisomal pathways were activated, while the metabolisms of some amino acids were suppressed (Fig. 4e). Nevertheless, none of these pathways were significantly altered (corrected *p* > 0.05).

**Fig. 5:**
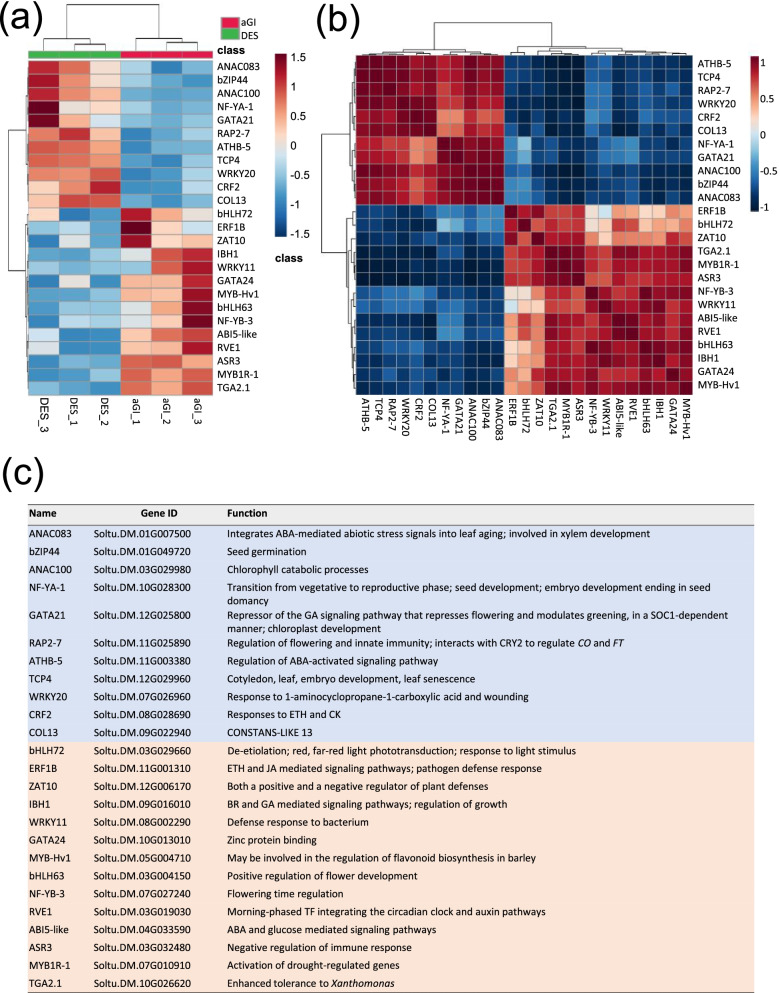
Differentially expressed transcription factors (TFs) in aGI52 leaves compared to the non-transformed control ‘Désirée’ (DES) leaves. **a** Heatmap. **b** Correlation heatmap. **c** List of differentially expressed TFs. Gene functions are based on UniProt (https://www.uniprot.org/) and PlantRegMap (http://plantregmap.gao-lab.org/). Abbreviation of hormones: ABA, abscisic acid; CK, cytokinin; ETH, ethylene; GA, gibberellic acid; JA, jasmonic acid

Transcription factors (TFs) are key regulators of plant development and stress responses. Thus, first we focused on differentially expressed TFs. Fourteen up-regulated and 11 down-regulated TFs were identified in aGI52 (Fig. 5a). The up-regulated category included the gene for bHLH63/CIB1, a positive regulator of flower development [11], NF-YB-3/HAP3C, which can interact with CO when it replaces HAP2 in the ternary HAP complex and promotes flowering in *Arabidopsis* [12], IBH1 that negatively regulates cell and organ elongation in response to gibberellin and brassinosteroid signalling [13], an *ABI5-like* gene similar to the major mediator of ABA repression of growth and floral transition in *Arabidopsis* [14, 15] and *RVE1,* a clock output affecting plant development [16]. Furthermore, TF genes involved in plant defence responses, *ERF1B*, *ZAT10*, *WRKY11*, *MYB1R1*, and *TGA2.1*, were also activated [17–21] in addition to *ASR3* that acts in an opposite and negative manner to regulate immune gene expression in *Arabidopsis* [22]. Interestingly, unlike *NF-YB-3/HAP3C*, *NF-YA-1/HAP2A*, encoding the DNA-binding subunit of the HAP complex, is down-regulated. *RAP2–7* encoding an ethylene responsive TF with an APETALA2 (AP2) domain is also down-regulated. AP2-like target genes act as floral repressors [23]. Furthermore, *GATA21*, a repressor of the gibberellin signalling pathway that also represses flowering [24], and a *CONSTANS-LIKE* gene, *COL13*, is expressed at lower levels in aGI52 than in DES. Figure 5b demonstrates that the expression of the majority of TFs listed in Fig. 5c are highly synchronised. The exceptions are *ZAT10*, *ERF1B*, and *bHLH72*, whose expression pattern is similar only to that of *TGA2.1*, *MYB1R-*1, and *ASR3*.

**Fig. 6:**
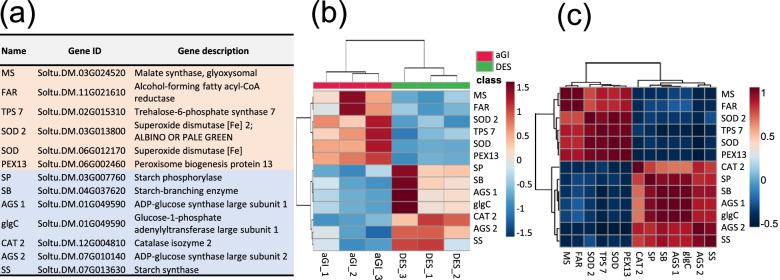
Differentially expressed genes in aGI52 leaves compared to the non-transformed control ‘Désirée’ (DES) leaves involved in glyoxylate and dicarboxylate metabolism, carbon metabolism and peroxisomal pathways. **a** List of differentially expressed genes. **b** Heatmap. **c** Correlation heatmap

The KEGG analysis highlighted some DEGs involved in glyoxylate and dicarboxylate metabolism, carbon metabolism, and peroxisomal pathways (Additional file 1: Fig. S8–10). These included the genes encoding the key enzymes of starch synthesis, *ADP-GLUCOSE SYNTHASE* (*AGS*), *STARCH SYNTHASE* (*SS*), and *STARCH PHOSPHORYLASE* (*SP*) in addition to *TREHALOSE-6-PHOSPHATE SYNTHASE* (*TPS*), a sugar messenger connecting metabolism and development, and the first line defence antioxidants, *SUPEROXIDE DISMUTASE* (*SOD*) and *CATALASE* (*CAT*) as shown in Fig. 6a. Nevertheless, while *SOD* was up-regulated, *CAT* was down-regulated (Fig. 6b). In contrast, down-regulation of starch synthesis genes was highly coordinated (Fig. 6c).

Although the anthocyinin content in leaves was so low that only cyanidin 3,5-di-O-glucoside could be detected the difference in the concentration of this compound between aGI52 (22.4 ± 0.2 ng/g Fw) and DES (24.1 ± 0.2 ng/g Fw) was significant (*p* < 0.01). This prompted us to make a manual search for DEGs involved in anthocyanin metabolism. Anthocyanins are a class of flavanoids synthesized through a branch of the phenylpropanoid pathway. The beginning genes include *PHENYLALANINE AMMONIA LYASE* (*PAL*), *CINNAMATE 4-HYDROXYLASE* (*C4H*), and *4-COUMARYOL COA LIGASE* (*4CL*). The next steps are divided into early and late enzymatic steps. The early biosynthetic genes include *CHALCONE SYNTHASE* (*CHS*), *CHALCONE ISOMERASE* (*CHI*), *FLAVANONE 3-HYDROXYLASE* (*F3H*), and *FLAVANONE 3′-HYDROXYLASE* (*F3’H*). The late biosynthesis genes include *DIHYDROFLAVONOL 4-REDUCTASE* (*DFR*), *ANTHOCYANIDIN SYNTHASE* (*ANS*), and *UDP-GLUCOSE:FLAVONOID 3-O-GLUCOSYL TRANSFERASE* (*UFGT*). A correlation between the expression of these genes and anthocyanin content of potato tubers was found. The pathway is transcriptionally regulated by the MBW complex consisting of MYB, bHLH, and WD40 TFs (reviewed in [25]). Nevertheless, none of the coding genes of the above listed proteins except *PAL* (log_2_ fold-change − 0.5) was differentially expressed in aGI52 leaves compared to DES leaves. Anthocyanin discoloration might be due to either lower level of synthesis or higher level of degradation. For anthocyanin degradation, the candidate gene families are *POLYPHENOL OXIDASES*, *PEROXIDASES*, and *β-GLUCOSIDASES* (reviewed in [25]). We found no change in expression of any of these genes in aGI52 although we found the *MYB-RELATED PROTEIN Hv1* (log_2_ fold-change 0.87) among the up-regulated TF genes (MYB-Hv1 in Fig. 5) that may be involved in the regulation of flavonoid biosynthesis and *LDOX* (log_2_ fold-change − 0.02), the coding gene of the enzyme oxidising leucoanthocyanidins into anthocyanidins, which was slightly, but significantly down-regulated in aGI52 leaves.

### Validation of RNA-seq data by real-time quantitative PCR

**Fig. 7:**
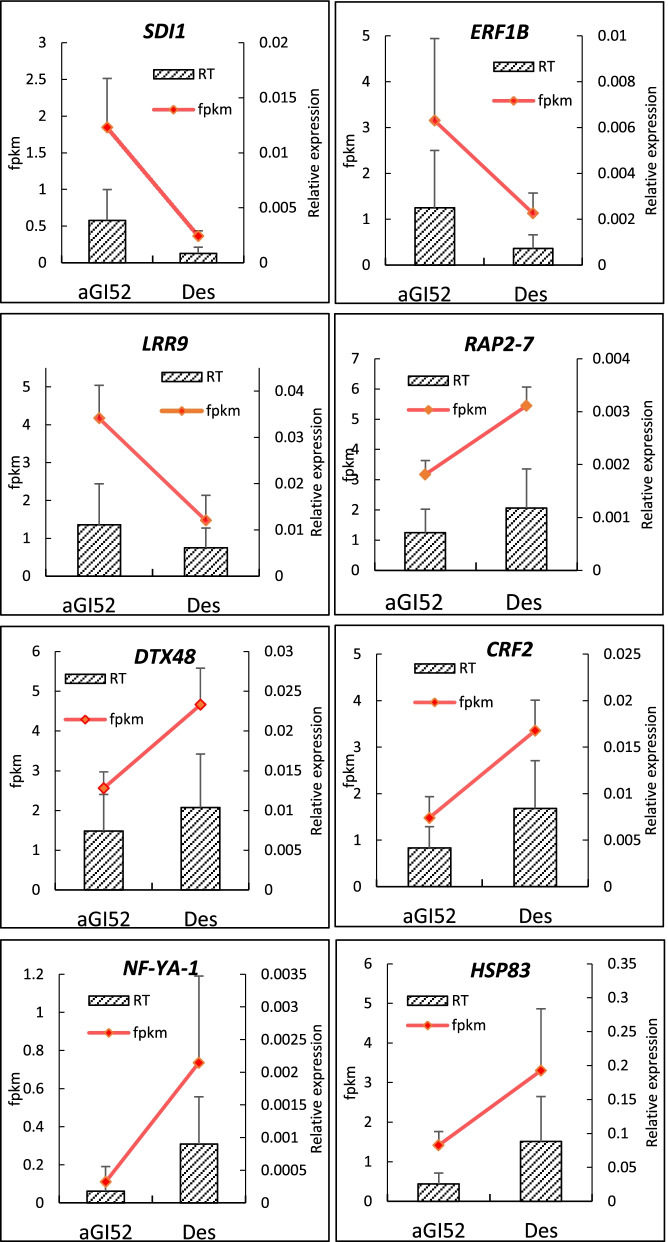
Expression of selected genes in aGI52 leaves compared to the non-transformed control ‘Désirée’ (DES) leaves detected by RNA-seq (red lines) and RT-qPCR (bars). Relative expression of the genes *SULFUR DEFICIENCY-INDUCED 1 - SD1* (Soltu.DM.06G002200), *ETHYLENE-RESPONSIVE TRANSCRIPTION FACTOR 1B - ERF1B* (Soltu.DM.11G001310)*, PUTATIVE F-BOX/LRR-REPEAT PROTEIN 9 - LRR9* (Soltu.DM.08G001370), *ETHYLENE-RESPONSIVE TRANSCRIPTION FACTOR - RAP2–7* (Soltu.DM.11G025890), *DETOXIFICATION 48 - DTX48* (Soltu.DM.04G031660), *ETHYLENE-RESPONSIVE TRANSCRIPTION FACTOR CRF2 - CRF2* (Soltu.DM.08G028690), *NUCLEAR TRANSCRIPTION FACTOR Y SUBUNIT A-1 - NF-YA-1* (Soltu.DM.10G028300) and *HEAT SHOCK PROTEIN 83 - HSP83* (Soltu.DM.06G006550) was established in comparison to the geometric mean expression values of *ACTIN* and *EF1α*. Error bars represent the standard deviation of three independent biological replicates. Each replicate contained 3 leaf discs of 1 cm in diameter harvested from 3 plants

To validate the results of RNA-seq data, we selected three significantly up-, and five down-regulated DEGs in aGI52. These genes included four TFs, a diagnostic indicator of sulphur nutritional status, an F-box/LRR-repeat protein, a transmembrane transporter, and a heat shock protein (Fig. 7). The expression trends of each selected gene using RT-qPCR and RNA-seq were similar, indicating that the transcriptome data are highly reliable.

### Phylogenetic and similarity analysis of StMYB-Hv1

**Fig. 8:**
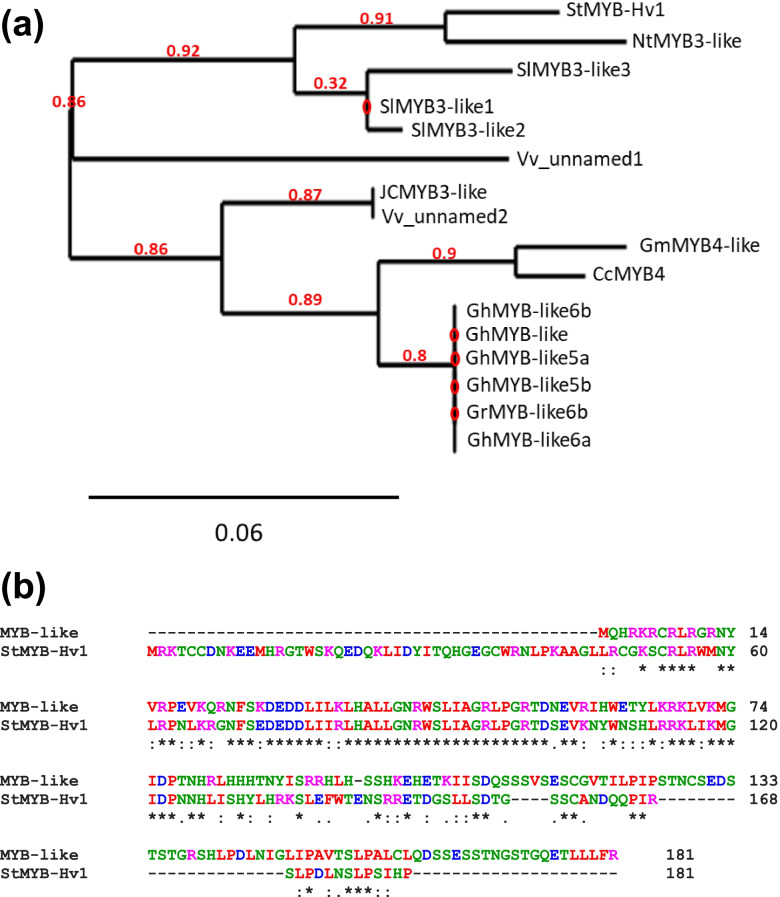
Phylogenetic tree (**a**) and alignment of StMYB-Hv1 and the *Arabidopsis* MYBL2 proteins. **b** Sequences producing significant alignments with StMYB-Hv1 (PGSC0003DMG400030548) were retrieved from iTAK and Phylogeny.fr was used to generate the tree. An NCBI protein-protein blast search was carried out and AtMYBL2 (NP_001321410.1) identified as the *Arabidopsis* protein with the highest similarity to StMYB-Hv1. Identities and similarities of sequences are presented as a result of a Clustal Omega multiple sequence alignment. St, *Solanum tuberosum*; Nt, *Nicotiana tomentosiformis*; Sl, *Solanum lycopersicum*; Vv, *Vitis vinifera*; JC, *Jatropha curcas*; Gm, *Glycine max*; Cc, *Cajanus cajan*; Gh, *Gossypium hirsutum*; Gr, *Gossypium raimondii*

MYB TFs are one of the largest TF families in plants which contain 1–4 tandem incomplete repeats (Rs) at the N-terminal region. According to the number of Rs the MYB family is divided into four categories (reviewed in [26]). It was found that StMYB-Hv1 is made up of 122 amino acids and belongs to the R3 MYB TFs carrying a single R3 type domain. The phylogenetic analysis showed that StMYB-Hv1 has the highest relationship with the MYB3-like proteins of *Nicotiana tomentosiformis* and *Solanum lycopersicum* (Fig. 8a and Additional file 1: Fig. S11). However, since the function of these proteins has not been known thus far a search for the most similar *Arabidopsis thaliana* protein sequence was carried out. This search resulted in identification of AtMYBL2 as the protein with the highest similarity to StMYB-Hv1 (Fig. 8b).

## Discussion

Despite detection of the pleiotropic function of GI in several plant species, only its role in tuber initiation was studied in potato. Based on the function of GI in other plant species, we supposed that it is not restricted to tuberisation in potato. To test this hypothesis, we repressed the expression of a *GI* gene in the commercial potato cultivar ‘Désirée’ and investigated the effect of repression at morphological and transcriptome level.

Recently, we have identified two *GI* genes in potato, designated *StGI.04* and *St.GI.12*, with different promoter sequences, different organ-specific expression, and stress responses [8]. Thus, we presumed that the two genes possess different functions at least in part. In this study, *StGI.04* was targeted and its mRNA level reduced by constitutive expression of its less homologous region to *St.GI.12* in antisense orientation. Nevertheless, even this region showed 71.2% identity with *St.GI.12* cDNA sequence. Although, no reduction in *St.GI.12* transcript level was detected by RT-qPCR due to the high level of homology existing between the two *GI* copies, the influence of *St.GI.12* on the results obtained cannot be excluded.

Reduction of *StGI.04* mRNA level in leaves and tubers of antisense aGI plants ranged between 2 and 51%. The most stable and relatively high level of repression (33–49%) was detected in aGI52. Thus, this line was studied in more detail than the others in our experiments.

Morphological characterisation of five aGI lines was carried out under greenhouse conditions. No differences in phenotype, growth rate, and tuber yield of the aGI plants compared to the control were detected. Although there was one-on-one line that showed differences in earliness of tuberisation or size distribution of tubers it was not in correlation with the level of *StGI.04* repression. Growing aGI52 plants under SD condition did not influence the tuberisation either. Earlier, we identified two POTH20 TF binding sites in the promoter region of *StGI.04* [8]. It was shown previously that overexpression of POTH1, a KNOTTED-like homeobox gene with 73% identity to POTH20, caused in vitro enhancement in tuberisation under both SD and LD photoperiods in several potato lines [27]. Therefore, we thought that the repression of *StGI.04* would result in alterations of tuber formation. However, this situation did not occur. Thus, we concluded that the level of reduction in the *StGI.04* transcript level that we could achieve might not be high enough for influencing tuberisation in the commercial potato cultivar ‘Désirée’.

The role of GI in photoperiodic flowering and circadian clock has been extensively investigated in several plant species, and it seems that flowering time regulation and circadian clock control are general GI functions. In line with this conclusion, binding sites for TFs involved in the regulation of flower development, including SOC1 and ABI5, were predicted, and expression of *StGI.04* in floral organs was demonstrated via RT-qPCR [8]. This study showed that *StGI.04* repression influences the expression of several TFs involved in regulation of flowering; the positive regulators of flowering *bHLH63/CIB1* and *ABI5-like* are up-regulated, while the repressors *RAP2–7* and *GATA21* are down-regulated in aGI52 leaves. The *CO-LIKE* gene, *COL13*, however, appears to be out of line because it is expressed at a lower level in aGI52 versus DES, while CO is an activator of flowering in *Arabidopsis* [28]. Although, we note that the function of COL13 has not been studied yet, and it can differ from that of CO. Repression of *StGI.04* produced up-regulation in the expression of *REVEILLE1* (*RVE1*), a Myb-like, clock-regulated TF that links the clock and auxin networks by positive regulation of the expression of the auxin biosynthetic gene *YUCCA8* (*YUC8*), thereby facilitating plant growth [16]. These results indicate that as in other plant species, GI is involved in circadian clock control.

Although both NF-YA-1/HAP2A and NF-YB-3/HAP3C are components of the NF-Y/HAP heterotrimer TF complex, *NF-YA-1/HAP2A* was down-regulated, while *NF-YB-3/HAP3C* was up-regulated in aGI52 leaves. In *Arabidopsis*, the NF-Y/HAP complex was shown to stimulate the transcription of various genes by recognizing and binding to a CCAAT motif in promoters. NF-Y complexes, among others, have been found to be involved in the control of flowering, embryogenesis, seed maturation, drought resistance, and ABA perception. The *Arabidopsis* genome encodes 10 distinct NF-YA, NF-YB, and NF-YC proteins that allow an enormous combinatorial and functional diversity for the complex (reviewed in [29]). The number of *NF-Y* genes may be even higher in the tetraploid potato than in *Arabidops*is, and the down-regulated NF-YA-1/HAP2A and the up-regulated NF-YB-3/HAP3C may be the subunits of different NF-Y/HAP complexes with different regulation pathways and functions.

Besides flowering, the role of GI in responses of plants to environmental stresses is also well-known. We found *StGI.04* to be induced by osmotic-, cold- and heat stresses [8] and identified five TF genes involved in plant defence responses (*ERF1B*, *ZAT10*, *WRKY11*, *MYB1R1, TGA2.1*), which were up-regulated in aGI52. ERF1 and WRKY11 led to an increase in tolerance to *Bacillus* species in *Arabidopsis* and tomato [17, 19], while the activity of TGA2.1 caused retardation in rice plants in defence against *Xanthomomas oryzae* [21]. A similar duality was observed in the case of ZAT10. Mittler et al. [18] found that both overexpression and knockout *zat10* mutants of *Arabidopsis* were enhanced in tolerance to salinity, heat, and osmotic stresses. In contrast, *MYB1R-1* caused an unambiguous increase in drought tolerance in potato [20]. After considering these literature data and our results, it is suggested that StGI.04 has an ambivalent role in terms of both the biotic and abiotic stress responses in potato.

Superoxide dismutase and catalase (SOD and CAT, respectively) are antioxidant enzymes and integral parts of plants’ defence mechanisms to avoid damage caused by active oxygen species. Interestingly, however, while *SOD* was up-regulated, *CAT* was down-regulated in aGI52 leaves. GI is a repressor of *SOD* also in *Arabidopsis* as it is indicated by enhanced tolerance of the *gi-3* mutant to oxidative stress, which is associated, at least in part, with constitutive activation of *SOD* and *APX* genes [30]. However, unlike in potato, the expression levels of *CAT* genes in *Arabidopsis* were 1.5–2-fold higher in the *gi* mutants than in the wild type [31].

We found that the key genes of starch synthesis, *AGS1*, *AGS2, SS*, and *SP* were suppressed, while *TPS*, a sugar messenger, was activated in aGI52. It has already been known that the *Arabidopsis* circadian system is sensitive to sucrose, the GI protein is stabilised by sucrose in the night and the *gi* mutants have increased starch contents [32–34]. Rice plants carrying a null mutation in *GI* showed a significantly increased sucrose and starch content in the leaves [35]. Our transcriptome result suggests that potato may be a third type of plants, in which repression of *StGI.04* decreases the starch content in leaves. A recent study identified TREHALOSE-6-PHOPHATE SYNTHASE 8 (TPS8) as a direct interactor of GI in *Arabidopsis* [36] and we found *TPS7* among the up-regulated genes. Our result supports the previous conclusion that TPS is involved in the mediation of GI effects.

Repression of *StGI.04* expression caused a reduction in tuber colour and the anthocyanin pigmentation of tuber skins. Significant reductions were detected in cyanidin 3,5-di-O-glucoside and pelargonidin 3,5-di-O-glucoside contents in tuber peels. Nevertheless, no significant alterations were found in the biosynthetic pathway of these compounds at the transcriptome level in leaves. The exceptions were *PAL,* the beginning gene of the pathway and *LDOX*, encoding a leucoanthocyanidin oxidising enzyme, which were down-regulated. The lack of transcriptional changes in leaves might be due to the general inactivity of the anthocyanin biosynthesis pathway, which is reflected by the very low amount of anthocyanin pigments detected in leaves. While the anthocyanin concentration was in a range from 30 to 430 μg/g Fw in tuber skin it was only 22–24 ng/g Fw in leaf samples and the only anthocyanin which could be detected in leaves was cyanidin 3,5-di-O-glucoside. However, its concentration in aGI52 leaves was slightly, but significantly lower than in DES leaves.

It has been known for a long time that three loci, *D* (developer), *R* (red), and *P* (purple) determines tuber colour. Jung et al. [37] demonstrated that the *D* locus encodes an R2R3 MYB TF, a part of the MYB-bHLH-WD40 complex, regulating anthocyanin synthesis. Several alleles of *R2R3 MYB*s as *StMYBA*1 and *StMYB113* were identified in cultivated tetraploid potatoes [38] and shown that an *R2R3-MYB* is a direct target of the small RNA regulation [39]. However, we did not find these *MYB* genes among the aGI52 DEGs. Instead, we found an R3-type MYB-coding gene up-regulated in aGI52 leaves, the *MYB-RELATED PROTEIN Hv1* (*MYB-Hv1*), similar to *MYBL2* in *Arabidopsis*. AtMYBL2 is a transcriptional repressor that negatively regulates anthocyanin biosynthesis [40]. We presume a similar function for MYB-Hv1 in potato and are going to study it in tuber skin.

**Fig. 9:**
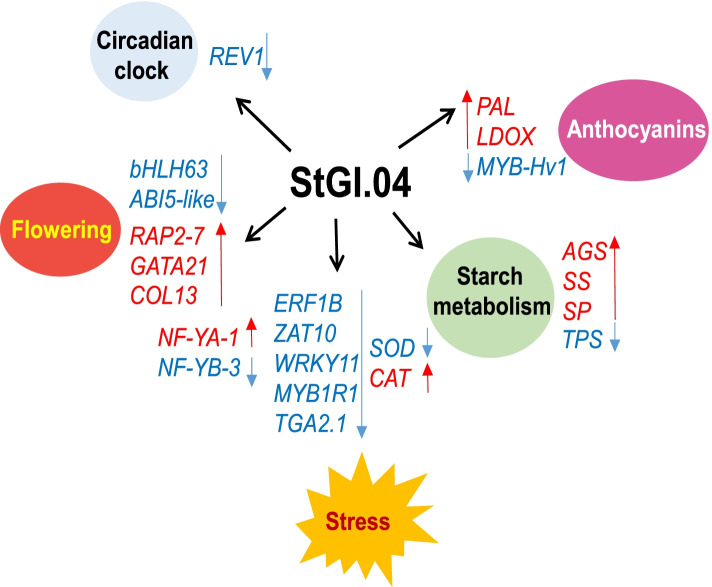
Schematic drawing presenting the biological processes and the major genes influenced by StGI.04 at transcript level. Up-regulated genes are in red, down-regulated genes are in blue

The biological processes, namely circadian clock regulation, flowering, stress responses, starch synthesis and anthocyanin metabolism and major genes influenced by StGI.04 at transcript level in potato leaves are presented in Fig. 9.

## Conclusions

*GIGANTEA* was discovered almost 60 years ago as a “supervital” *Arabidopsis* mutant with a late flowering phenotype [41]. Since that time, it has been detected in many plant species, and its pleiotropic effect extending from flowering through metabolism to stress responses has been demonstrated (reviewed in [3]). In most of the species, *GI* is a single copy gene; however, it is duplicated in petunia and potato [8, 42]. In this study, functional analysis of one of the potato *GI* genes, designated *StGI.04*, was performed. Analysis of a *StGI.04*-repressed line demonstrated that *StGI.04*, as with *GI* in other plant species, influences the circadian clock, flowering, stress responses, and starch synthesis via the alteration of expression of key genes of these processes in leaves of potato plants (Fig. 9). In addition, however, *StGI.04* has a new function not detected earlier in other plant species, namely promotion of the synthesis of anthocyanins in tuber skin. The function of the other potato *GI* gene, *StGI.12*, is still unknown. However, based on the differences in regulation of *StGI.04* and *StGI.12* [8], functional differences between the two potato *GI* genes are expected.

## Methods

### Plant growth conditions

The commercial potato (*Solanum tuberosum* L.) cultivar ‘Désirée’ was used in all experiments. It was obtained from Fritz Lange KG (Bad Schwartau, Germany), cultivated under axenic conditions for tissue culture at the Max Planck Institute of Molecular Plant Physiology (Golm, Germany) as described by Dietze et al. [43], and transferred to our laboratory. The plants were propagated in vitro from stem segments in the rooting medium RM (MS without vitamins [44]) containing 2% (w/v) sucrose and 0.8% agar in 40-ml tubes closed with paper plugs at 24 °C under a photoperiod cycle of 16 h/8 h day/night at a light intensity of 75 μmol m^− 2^ s^− 1^.

For morphological studies, 4-week-old plantlets obtained by tissue culture in tubes were transferred into pots containing Tabaksubstrat sterile soil A200 (Stender GmbH, Schermbeck, Germany) and grown further under greenhouse conditions from January to April at a temperature regime of 18 to 24 °C and soil humidity up to approximately 80% provided by regular watering. The photoperiod was set at 12 h light and 12 h dark. In winter, the ambient light conditions were supplemented with artificial lightening by sodium lamps. Pesticides and fungicides were regularly applied for pest and fungal pathogen control.

For the short day (SD) tuberisation experiment, plantlets transferred from in vitro into pots were grown in the greenhouse for a month and then moved into a growth chamber and grown further until the end of the vegetation period under well-controlled SD conditions (8 h light/16 h dark, 23 °C, 100 μmol m^− 2^ s^− 1^ light intensity, 72% humidity).

### Generation of *aGI*-repressed ‘Désirée’ lines

Antisense *GIGANTEA* (aGI) plants were generated by amplifying a 250-bp fragment of *StGI.04* using the primer pair GI250 (Additional file 2: Table S2) from ‘Désirée’ genomic DNA isolated according to Shure et al. [45]. The PCR fragment was inserted into pGEM-T Easy (Promega, Madison, WI, USA) and re-cloned in antisense orientation into the binary vector pCP60 as a *Kpn*I-*Eco*RI- fragment behind the constitutive *CaMV35S* promoter. pCP60 (Additional file 1: Fig. S12) is a derivative a pBIN19 [46] constructed and was generously provided by P. Ratet, CNRS, Paris, France. The recombinant plasmid DNA was transformed into *Escherichia coli* DH5α and introduced into *Agrobacterium tumefaciens* LBA4404 by tri-parental mating [47]. Identity and orientation of the 250-bp *StGI.04* fragment in pCP60 was verified by Sanger sequencing (BIOMI Ltd., Gödöllő, Hungary).

‘Désirée’ leaves propagated in vitro in 500-ml jars in MS medium containing 2% (w/v) sucrose and solidified with 0.8% agar (5 plants/jars) were used for transformation, and shoots were regenerated as described by Dietze et al. [43]. Kanamycin in a concentration of 25 μg ml^− 1^ was used for selection of transgenic lines.

### Analysis of *StGI* gene expression

Total RNA was extracted from leaves and tubers using the method described previously by Stiekema et al. [48]. RNA concentration and quality were tested using a NanoDrop spectrophotometer and reverse transcribed into first-strand cDNA using the Maxima H minus First Strand cDNA Synthesis Kit with dsDNase (Thermo Scientific Molecular Biology, Waltham, MA, USA). Semi-quantitative reverse transcription PCR (RT-PCR) analysis of the cDNAs was carried out using the ACTIN, GI.04spec and GI.12spec primer pairs (Additional file 2: Table S2) and visualising the PCR products on agarose gels. For these PCR amplifications, DreamTaq DNA Polymerase (Thermo Fisher Scientific, Waltham, MA, USA) was used.

RT-qPCR assays were performed using a Light Cycler-96 thermal cycler (Roche Diagnostics GmbH, Mannheim, Germany) and a Luminaris Color HiGreen Flourescein qPCR Master Mix (Thermo Scientific Molecular Biology, Waltham, MA, USA). *ACTIN* and *EF1α* served as reference genes [49]. Primers are listed in Additional file 2: Table S2.

### RNA-seq

RNA was isolated according to Stiekema et al. [48] from a sample set of three source leaves harvested from three 8-week-old plants grown in pots in the greenhouse. Three sample sets were prepared from aGI52, and three sets were prepared from the non-transformed control ‘Désirée’. A total of 5 to 7.5 μg of RNA per sample set was transported to the Novogene (UK) Company Ltd. (Cambridge, UK) and used for the generation of sequencing libraries after quality control. A paired-end 150 bp sequencing strategy was used to sequence the samples and the resulting data was also checked for its quality by the company. The bioinformatic analysis including mapping of quality reads to the *S. tuberosum* group Phureja DM1–3 v.6.1 reference genome (http://solanaceae.plantbiology.msu.edu/dm_v6_1_download.shtml), gene expression level analysis (FPKM distribution, Pearson correlation between samples, coexpression Venn diagram), differential gene expression (log2 fold change of DEGs, Vulcano plot) and functional analyses (GO and KEGG enrichment analyses) was carried out also by the company Novogene.

### Spectrophotometric analysis of anthocyanins

Freshly harvested, mature potato tubers grown in pots were peeled. A simplified method of Toguri et al. [50] was used for anthocyanin extraction from 1 g skin with 10 ml 1% HCl in methanol overnight at 4 °C. Relative concentrations of the chloride forms of the anthocyanin pigments were determined spectrophotometrically by measuring the absorbance at 540 nm.

### HPLC analysis of anthocyanins

The anthocyanin profile of potato tuber skin was analysed by the modified method on the basis of our previous studies [51–53]. The anthocyanin extract of tuber skin was centrifuged in Eppendorf tubes in a Hettich Mikro 22R centrifuge (15,000 rpm min^− 1^ for 2 min). The supernatant of centrifuged extract was filtered on a 0.45 μm MILLEX®-HV Syringe Driven Filter Unit (SLHV 013 NL, PVDF Durapore), purchased from Millipore Co. (Bedford, MA, USA), and injected into the HPLC system. The WATERS® High Performance Liquid Chromatograph (Waters Co., 34 Maple Street, Milford, MA, USA) was equipped with an absorbance detector (2487 Dual λ), a binary HPLC pump (1525), and in-line degasser, a column thermostat and an autosampler (717 plus) (set at 5 °C) and was controlled using EMPOWER™ 2 software. A SYMMETRY C18 5 μm 4.6 × 150 mm (pore size 100 Å) column was installed. The isocratic flow rate of mobile phase (2.5% glacial acetic acid containing Milli-Q® water, MeOH and ACN = 35:5:10) was set to 1 cm^3^ min^− 1^ the pressure in the column was 1600 ± 15 psi (cca 11 ± 0.1 MPa) at a column temperature of 40 °C. The running time was 10 min. The injected volumes of standards and samples were 20 μl. Detection started at 0.80 min to eliminate the peak of the extraction mixture, especially MeOH. The sampling rate was 10 pt. sec^− 1^, and the anthocyanins were monitored at a wavelength of 530 nm. The retention times of the standards were cyanidin 3,5-di-O-glucoside chloride (cyanin chloride) (CAS number: 2611–67–8) 1.6 min, pelargonidin 3,5-di-O-glucoside chloride (pelargonin chloride) (CAS number: 17334–58–6) 3.3 min, delphinidin 3,5-di-glycoside chloride (delphin) (CAS number: 17670–06-3) 4.3 min, and malvidin 3-galactoside chloride (primulin) (CAS number: 30113–37–2) 5.8 min.

### Phylogenetic and sequence similarity analysis

DNA and protein sequences were aligned with the NCBI nucleotide and protein blast (https://blast.ncbi.nlm.nih.gov/Blast.cgi) or with Clustal Omega (https://www.ebi.ac.uk/Tools/msa/clustalo/). Plant Transcription Factor and Protein Kinase Identifier and Classifier (iTAK; http://itak.feilab.net/cgi-bin/itak/index.cgi) was used to search for proteins with significant similarity. The phylogenetic tree was constructed using Phylogeny.fr (https://www.phylogeny.fr/). All analyses were carried out with default parameters.

### Statistical analysis

Heatmaps were generated by the Metaboanalyst 5.0 (https://www.metaboanalyst.ca). Statistical significance of the measurements was determined by Student’s *t*-test.

## Supplementary Information

**Additional file 1: Figure S1.** Nucleotide sequence of the *StGI.04* fragment used to generate the aGI lines and its alignment to the corresponding region of *StGI.12*. **Figure S2.** Growth and tuberisation parameters of *aGI*-repressed lines compared to the non-transformed DES control. **Figure S3.** Morphology of mature tubers collected from plants grown in a greenhouse in pots. **Figure S4.** FPKM distributions. **Figure S5.** Heatmap of the correlation coefficients between samples. **Figure S6.** Vulcano plot for differentially expressed genes. **Figure S7.** GO functional classification. **Figure S8.** KEGG pathway of glyoxalate and dicarboxylate metabolism showing the up- and down-regulated genes in aGI52 leaves. **Figure S9.** KEGG pathway of starch and sucrose metabolism showing the up- and down-regulated genes in aGI52 leaves. **Figure S10.** KEGG pathway of peroxisome showing the up- and down-regulated genes in aGI52 leaves. **Figure S11.** Sequence aligment of StMYB-Hv1-related proteins. **Figure S12.** Map of the binary vector pCP60.

**Additional file 2: Table S1.** RNA-seq quality statistics. **Table S2.** Primer sequences.

## Data Availability

The raw RNA-seq data has been submitted to GEO (https://www.ncbi.nlm.nih.gov/geo/info/linking.html.) under the Series record: GSE189956. All other data generated or analysed during this study are included in this published article and its supplementary information files.

## Abbreviations

DEG: Differentially expressed genes
DES: Désirée
GI: GIGANTEA
KEGG: Kyoto encyclopedia of genes and genomes
LD: Long day
SD: Short day
*St*: *Solanum tuberosum*
RT-qPCR: Reverse transcription-quantitative polyamerase chain reaction
TF: Transcription factor
