# Supplementary material for: Effects of the repression of GIGANTEA gene StGI.04 on the potato leaf transcriptome and the anthocyanin content of tuber skin
Source: BMC Plant Biol. 2022 May 20;22:249. doi: 10.1186/s12870-022-03636-3 (PMC9121593; doi:10.1186/s12870-022-03636-3)
Supplement: Supplementary file 1 — Additional file 1: Figure S1. Nucleotide sequence of the StGI.04 fragment used to generate the aGI lines and its alignment to the corresponding region of StGI.12. Figure S2. Growth and tuberisation parameters of aGI-repressed lines compared to the non-transformed DES control. Figure S3. Morphology of mature tubers collected from plants grown in a greenhouse in pots. Figure S4. FPKM distributions. Figure S5. Heatmap of the correlation coefficients between samples. Figure S6. Vulcano plot for differentially expressed genes. Figure S7. GO functional classification. Figure S8. KEGG pathway of glyoxalate and dicarboxylate metabolism showing the up- and down-regulated genes in aGI52 leaves. Figure S9. KEGG pathway of starch and sucrose metabolism showing the up- and down-regulated genes in aGI52 leaves. Figure S10. KEGG pathway of peroxisome showing the up- and down-regulated genes in aGI52 leaves. Figure S11. Sequence aligment of StMYB-Hv1-related proteins. Figure S12. Map of the binary vector pCP60. [file 12870_2022_3636_MOESM1_ESM.pptx]

## Slide 1
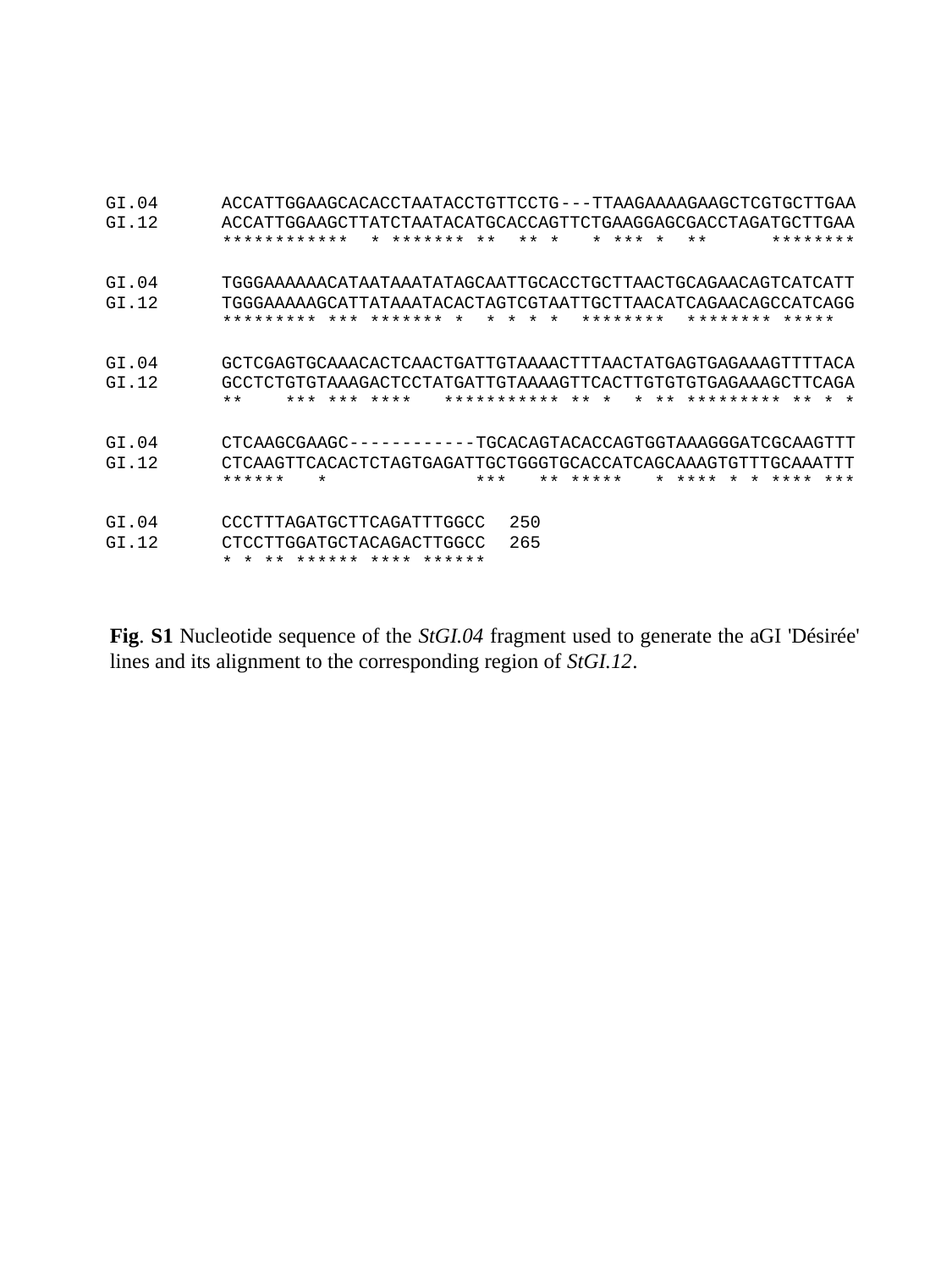

Fig. S1 Nucleotide sequence of the StGI.04 fragment used to generate the aGI 'Désirée' lines and its alignment to the corresponding region of StGI.12.

## Slide 2
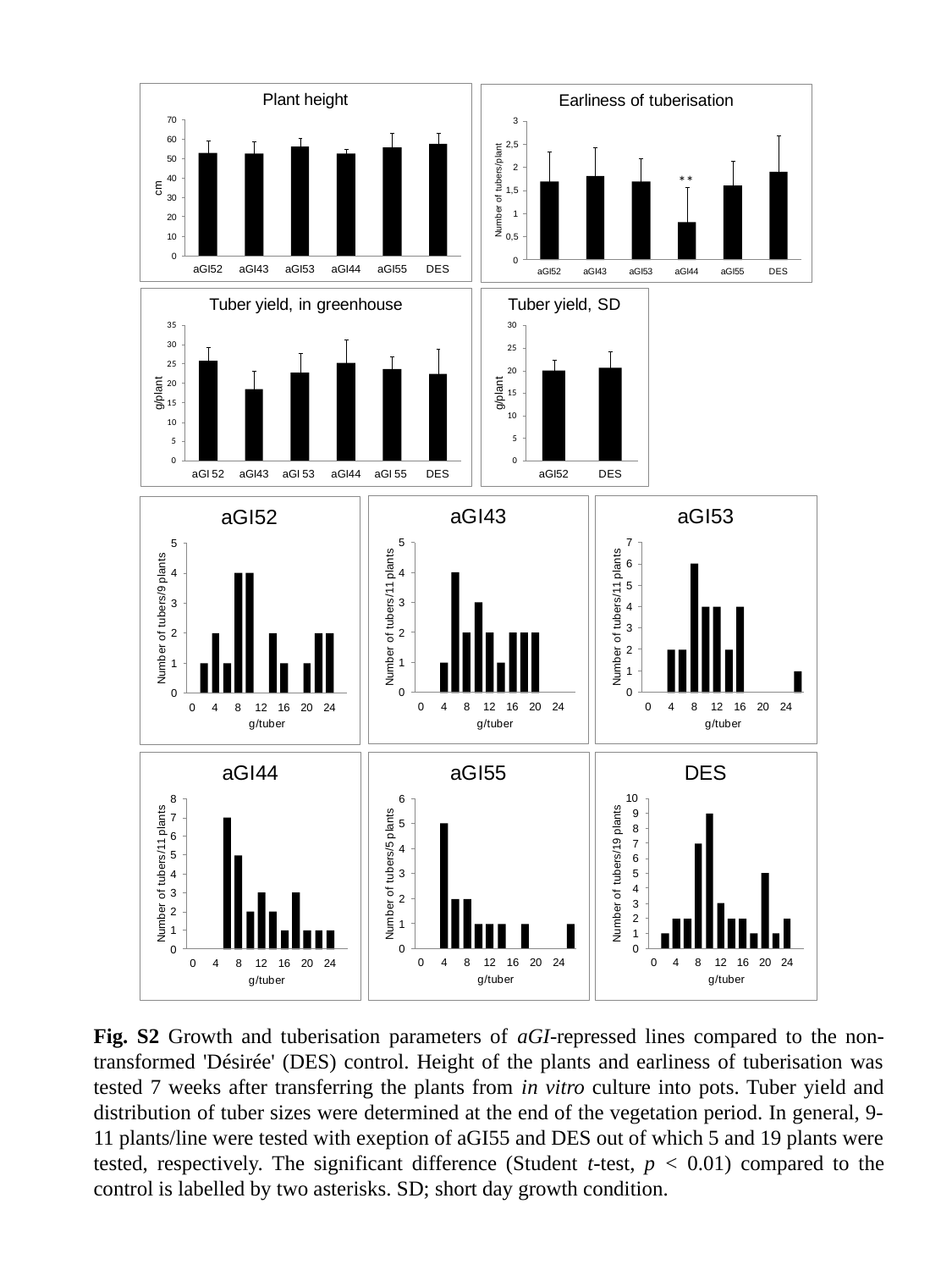

**
Fig. S2 Growth and tuberisation parameters of aGI-repressed lines compared to the non-transformed 'Désirée' (DES) control. Height of the plants and earliness of tuberisation was tested 7 weeks after transferring the plants from in vitro culture into pots. Tuber yield and distribution of tuber sizes were determined at the end of the vegetation period. In general, 9-11 plants/line were tested with exeption of aGI55 and DES out of which 5 and 19 plants were tested, respectively. The significant difference (Student t-test, p < 0.01) compared to the control is labelled by two asterisks. SD; short day growth condition.

## Slide 3
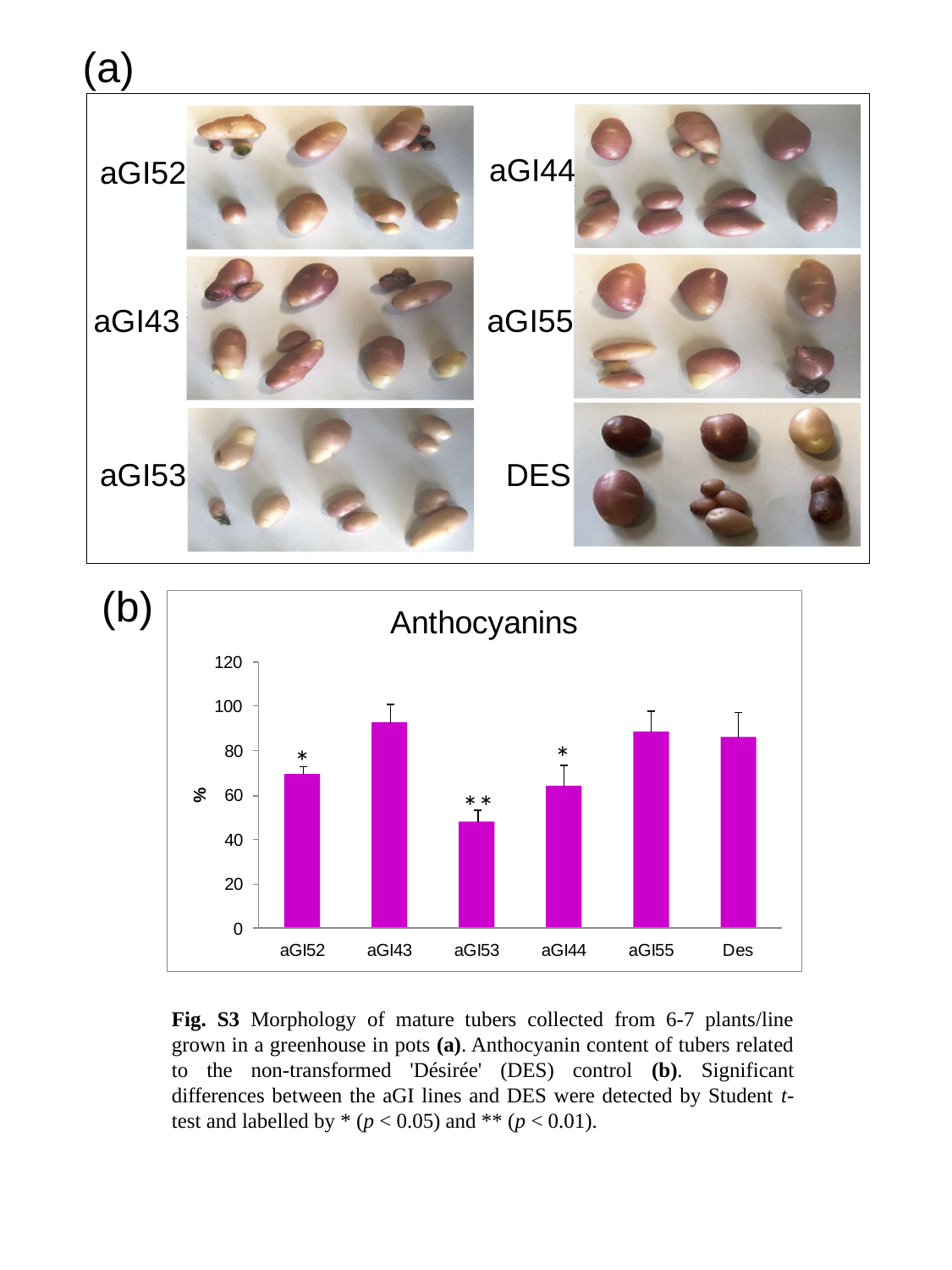

(a)
aGI52
aGI43
aGI53
aGI44
aGI55
DES
(b)
*
*
**
Fig. S3 Morphology of mature tubers collected from 6-7 plants/line grown in a greenhouse in pots (a). Anthocyanin content of tubers related to the non-transformed 'Désirée' (DES) control (b). Significant differences between the aGI lines and DES were detected by Student t-test and labelled by * (p < 0.05) and ** (p < 0.01).

## Slide 4
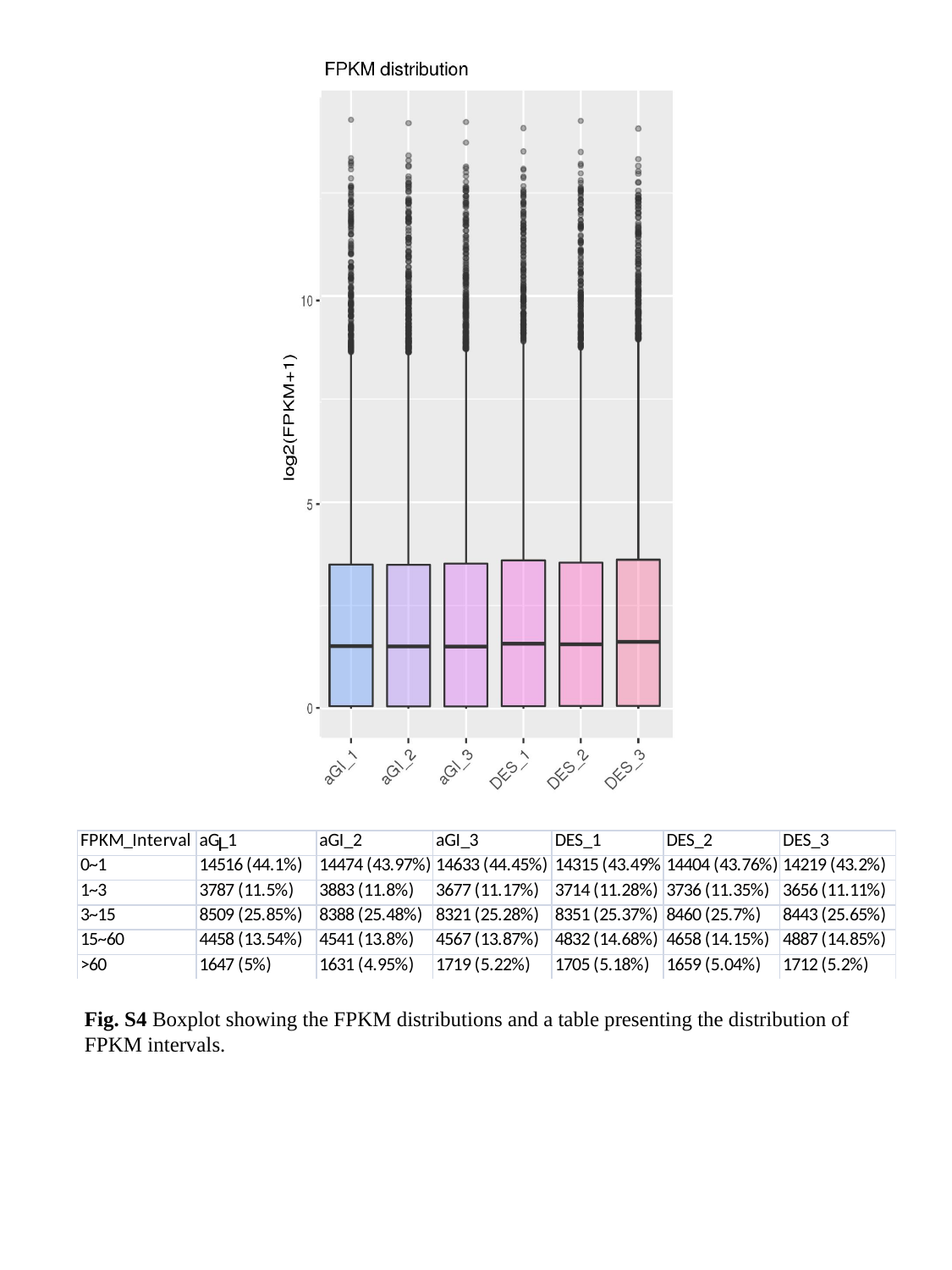

I
Fig. S4 Boxplot showing the FPKM distributions and a table presenting the distribution of FPKM intervals.

## Slide 5
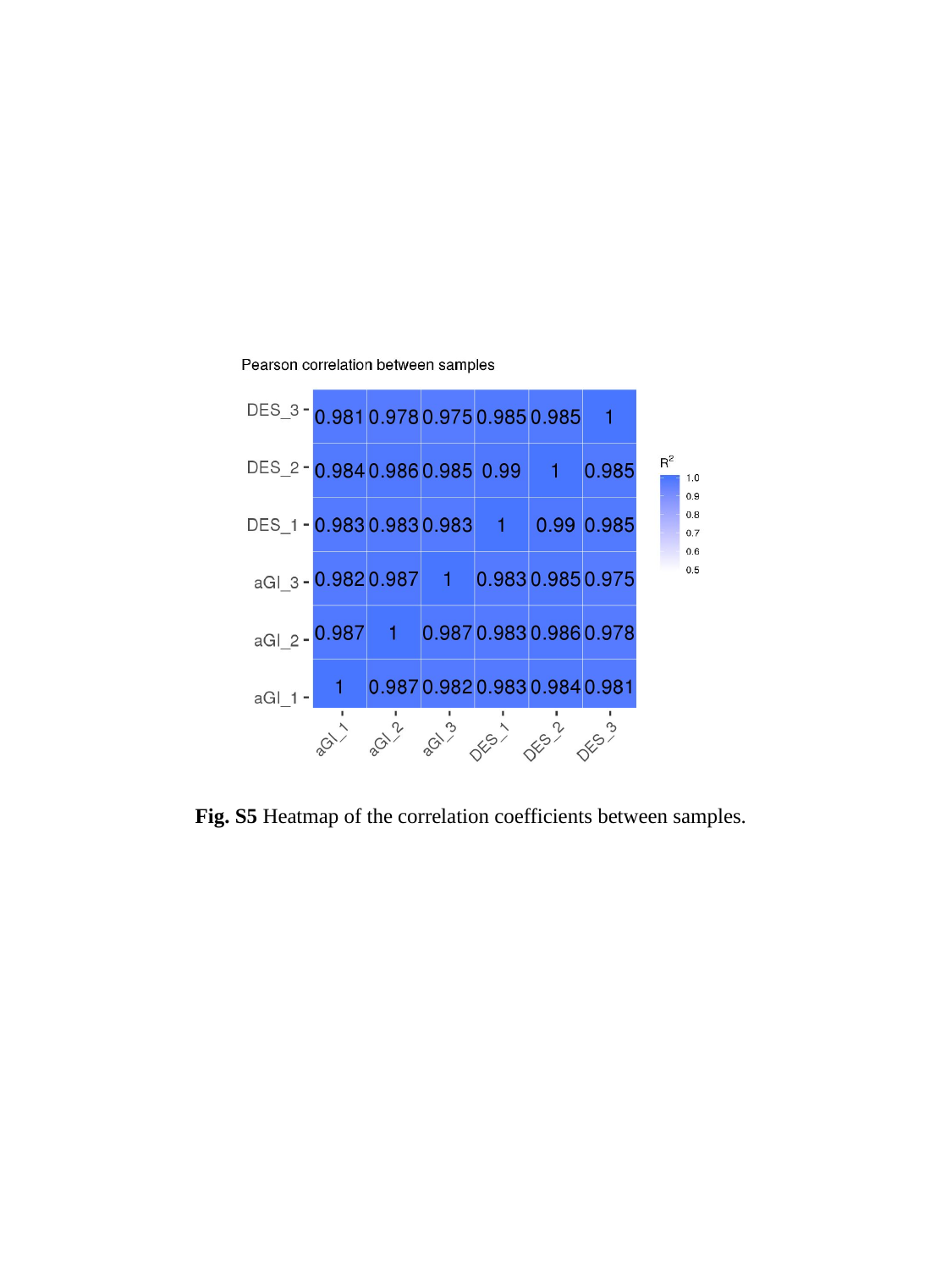

Fig. S5 Heatmap of the correlation coefficients between samples.

## Slide 6
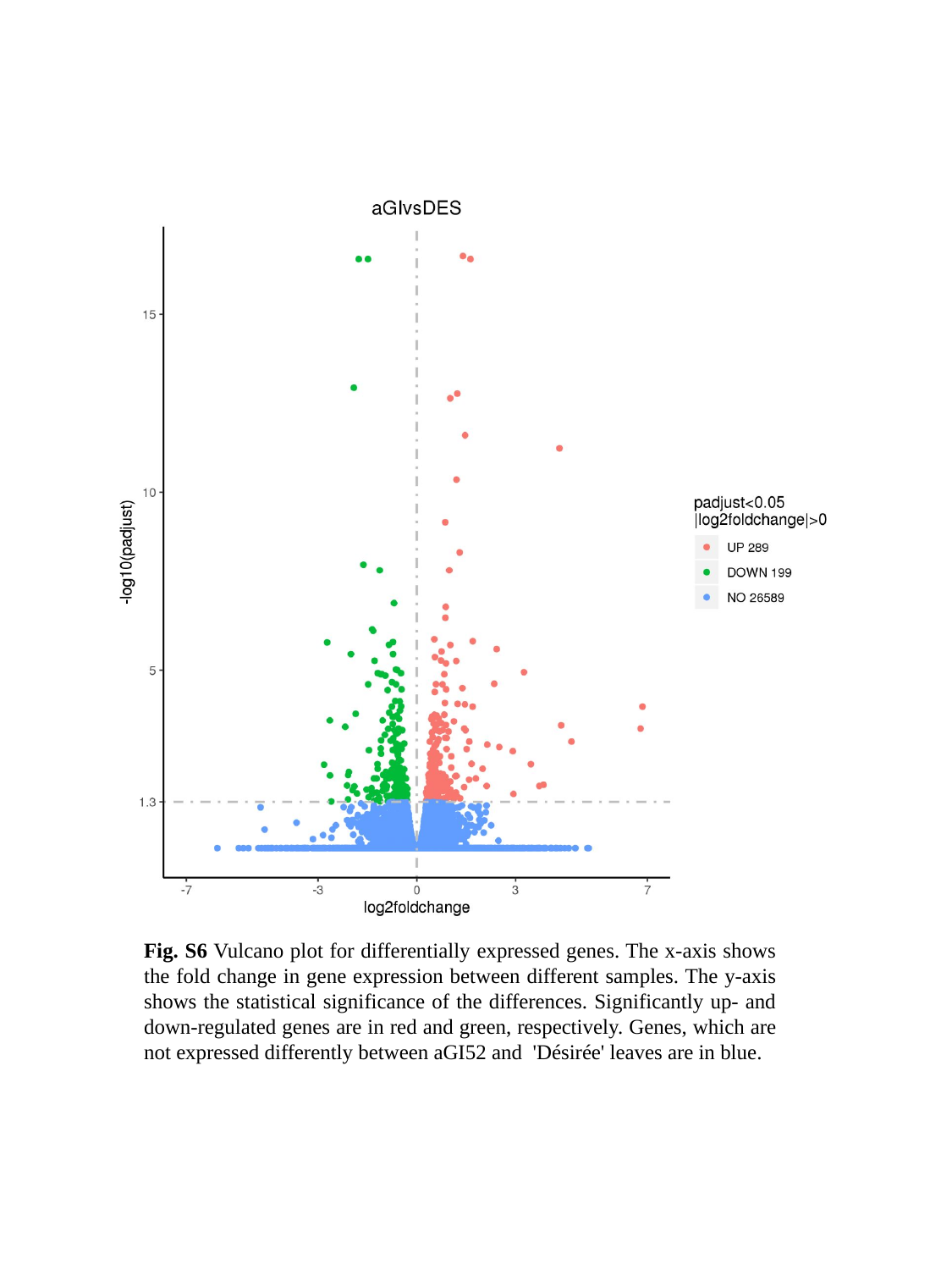

Fig. S6 Vulcano plot for differentially expressed genes. The x-axis shows the fold change in gene expression between different samples. The y-axis shows the statistical significance of the differences. Significantly up- and down-regulated genes are in red and green, respectively. Genes, which are not expressed differently between aGI52 and 'Désirée' leaves are in blue.

## Slide 7
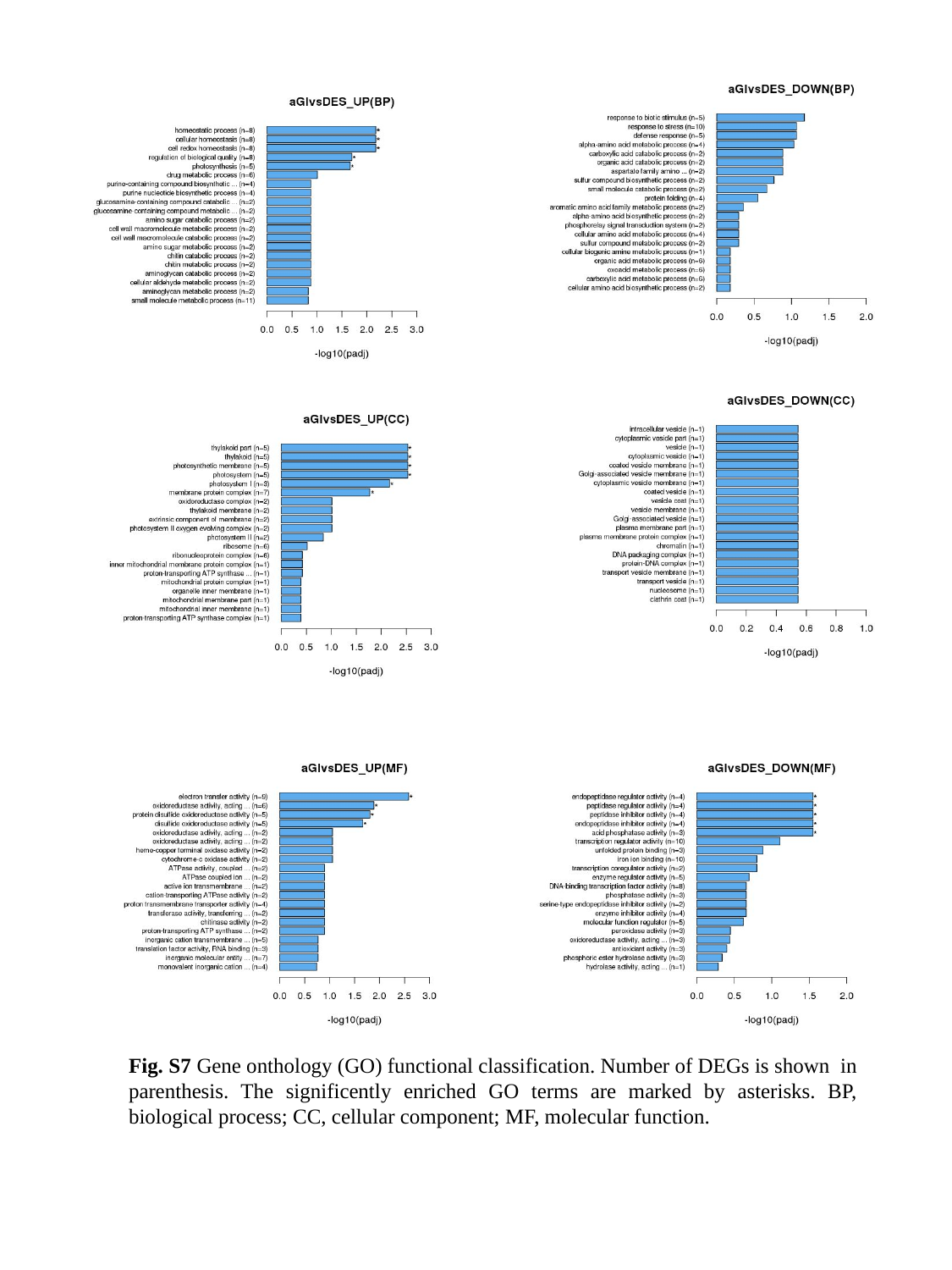

Fig. S7 Gene onthology (GO) functional classification. Number of DEGs is shown in parenthesis. The significently enriched GO terms are marked by asterisks. BP, biological process; CC, cellular component; MF, molecular function.

## Slide 8
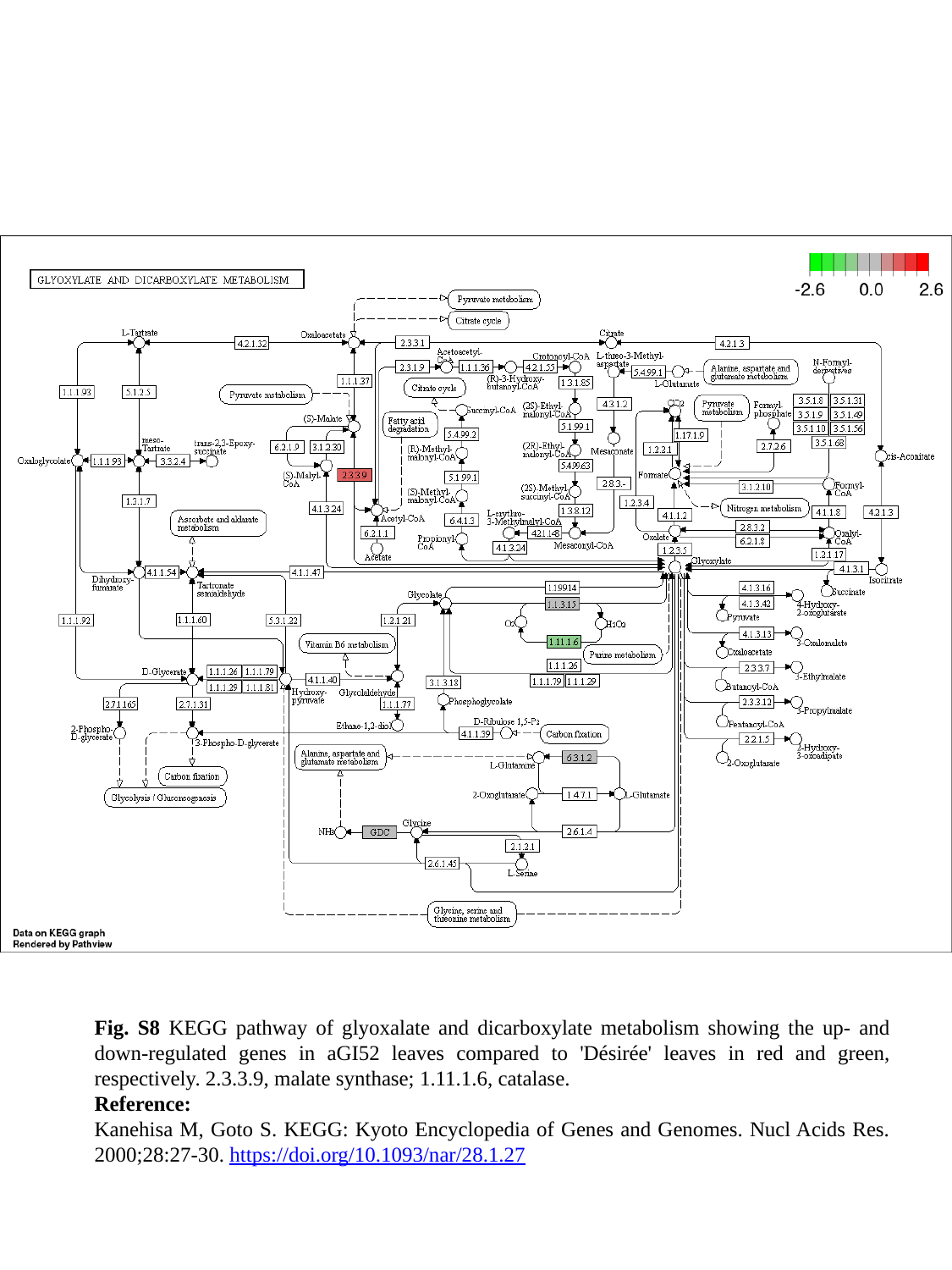

Fig. S8 KEGG pathway of glyoxalate and dicarboxylate metabolism showing the up- and down-regulated genes in aGI52 leaves compared to 'Désirée' leaves in red and green, respectively. 2.3.3.9, malate synthase; 1.11.1.6, catalase.
Reference:
Kanehisa M, Goto S. KEGG: Kyoto Encyclopedia of Genes and Genomes. Nucl Acids Res. 2000;28:27-30. https://doi.org/10.1093/nar/28.1.27

## Slide 9
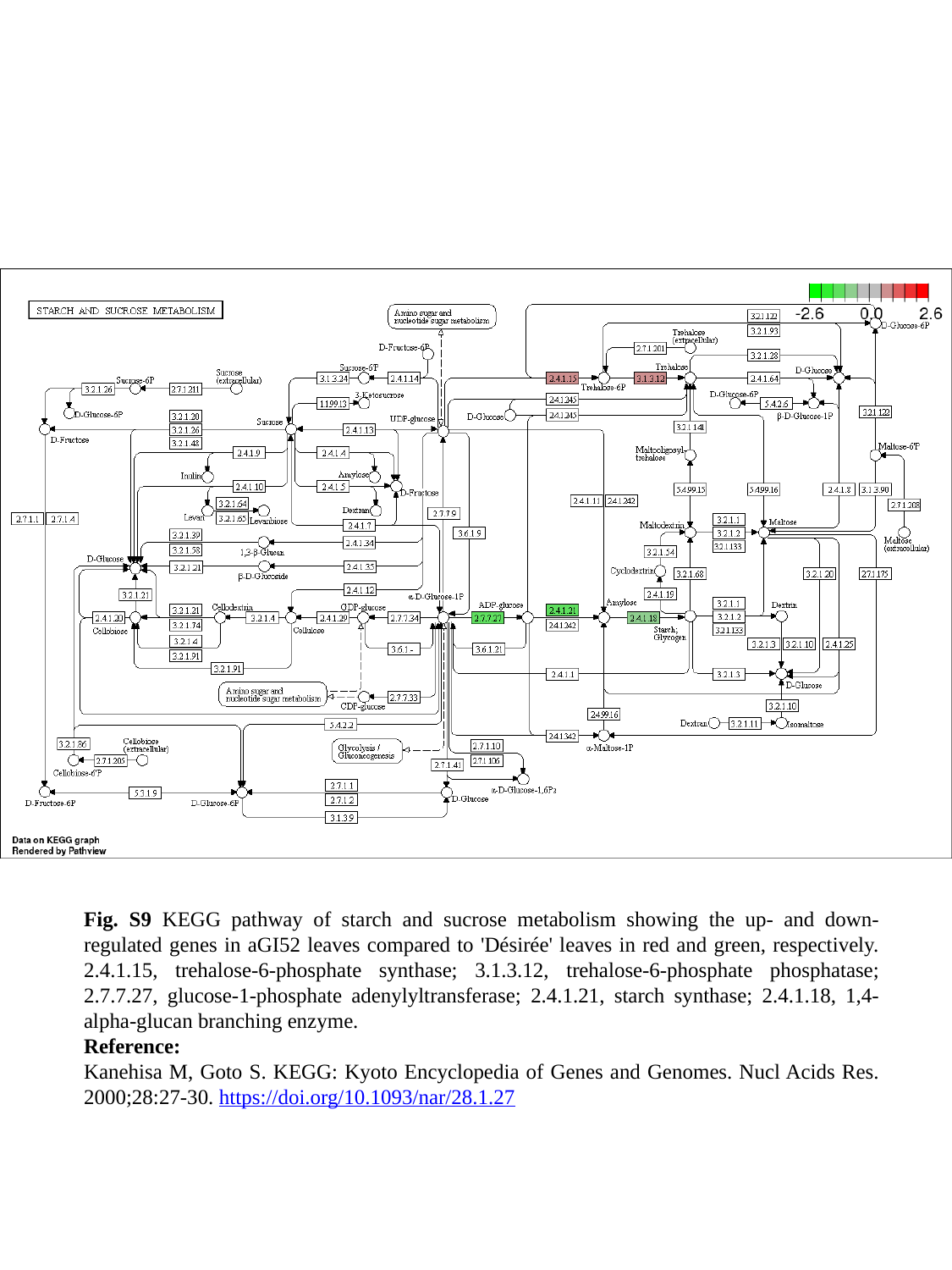

Fig. S9 KEGG pathway of starch and sucrose metabolism showing the up- and down-regulated genes in aGI52 leaves compared to 'Désirée' leaves in red and green, respectively. 2.4.1.15, trehalose-6-phosphate synthase; 3.1.3.12, trehalose-6-phosphate phosphatase; 2.7.7.27, glucose-1-phosphate adenylyltransferase; 2.4.1.21, starch synthase; 2.4.1.18, 1,4-alpha-glucan branching enzyme.
Reference:
Kanehisa M, Goto S. KEGG: Kyoto Encyclopedia of Genes and Genomes. Nucl Acids Res. 2000;28:27-30. https://doi.org/10.1093/nar/28.1.27

## Slide 10
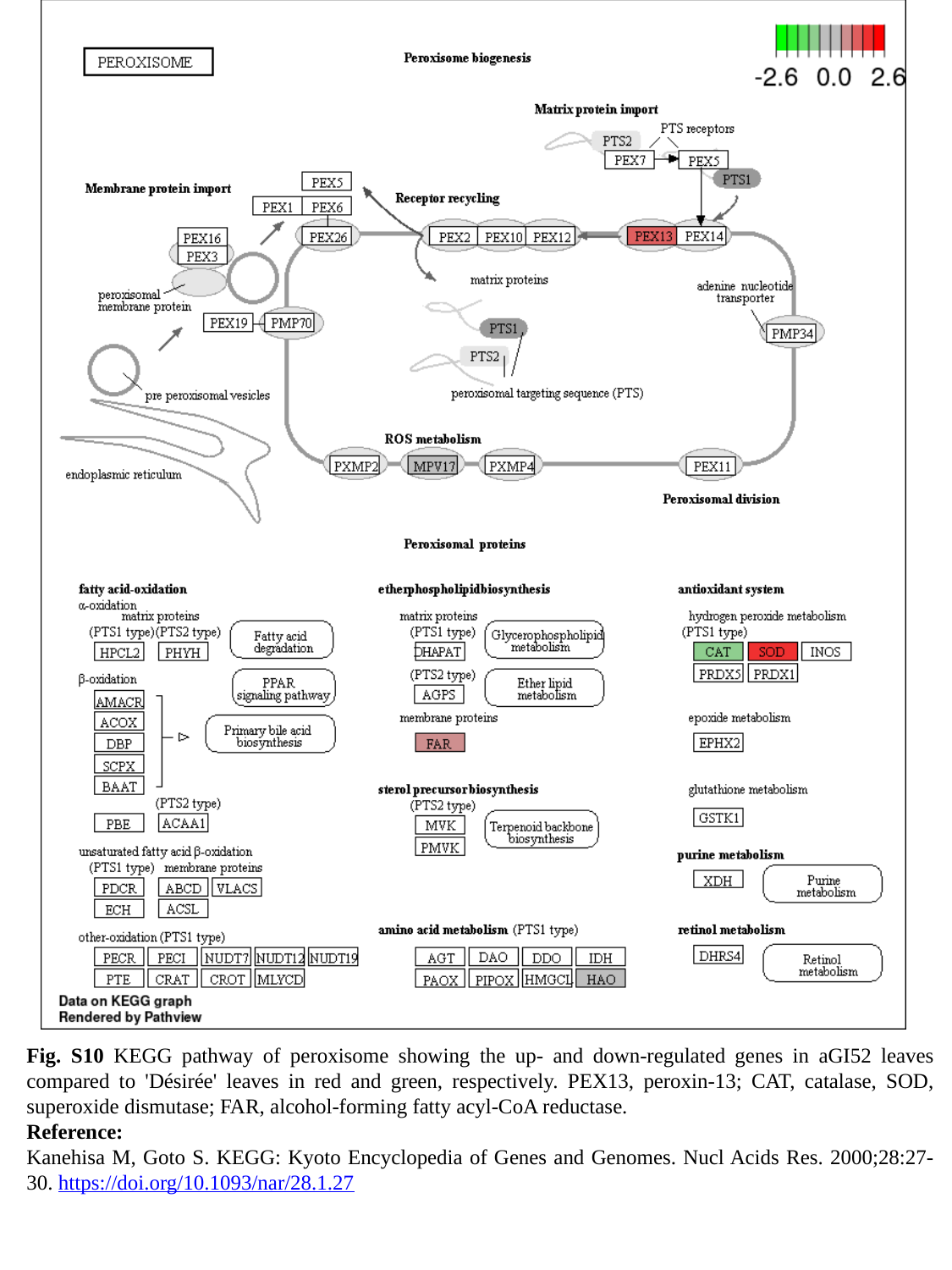

Fig. S10 KEGG pathway of peroxisome showing the up- and down-regulated genes in aGI52 leaves compared to 'Désirée' leaves in red and green, respectively. PEX13, peroxin-13; CAT, catalase, SOD, superoxide dismutase; FAR, alcohol-forming fatty acyl-CoA reductase.
Reference:
Kanehisa M, Goto S. KEGG: Kyoto Encyclopedia of Genes and Genomes. Nucl Acids Res. 2000;28:27-30. https://doi.org/10.1093/nar/28.1.27

## Slide 11
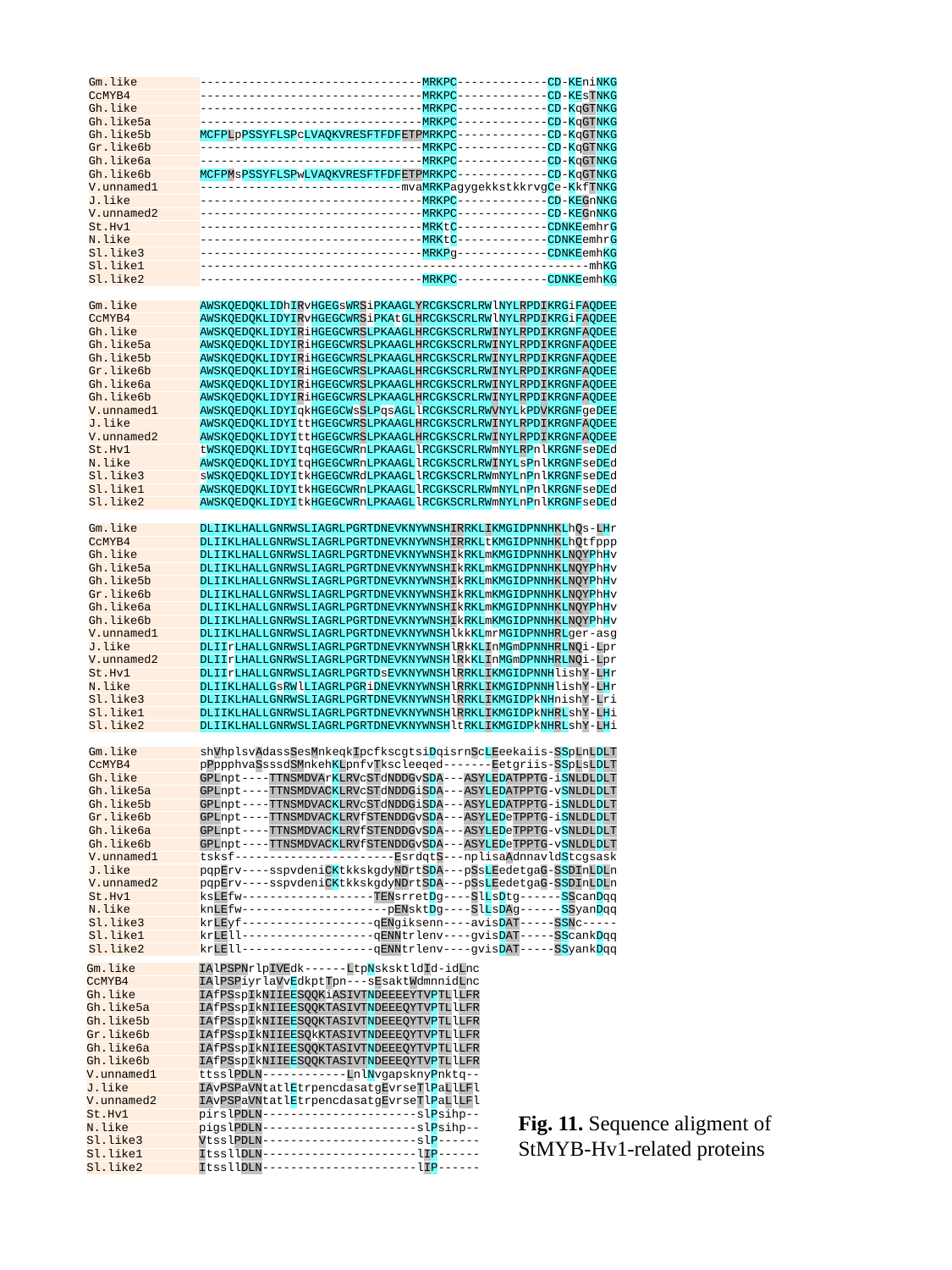

Fig. 11. Sequence aligment of
StMYB-Hv1-related proteins

## Slide 12
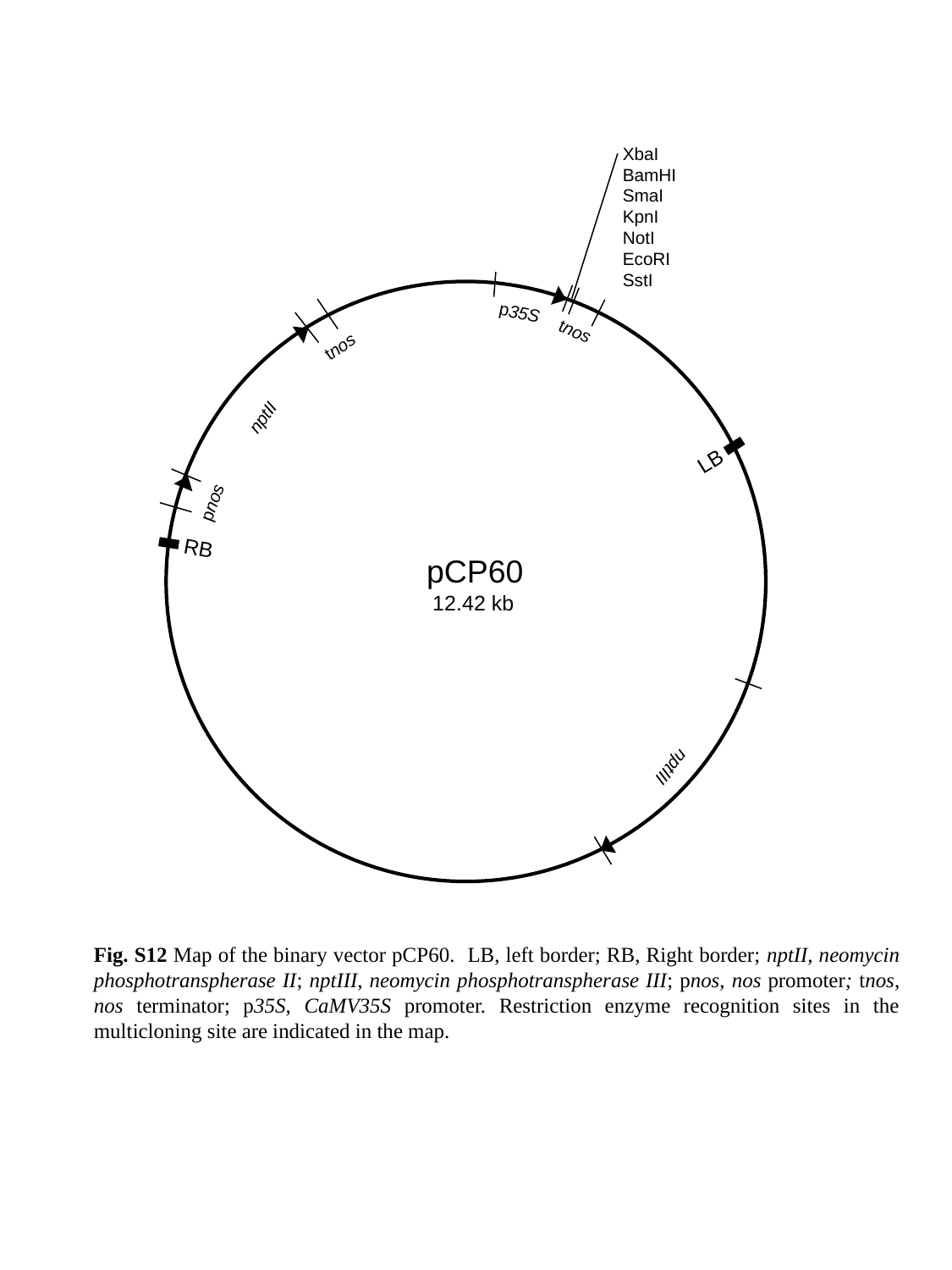

XbaI
BamHI
SmaI
KpnI
NotI
EcoRI
SstI
p35S
tnos
tnos
nptII
LB
pnos
RB
pCP60
 12.42 kb
nptIII
Fig. S12 Map of the binary vector pCP60. LB, left border; RB, Right border; nptII, neomycin phosphotranspherase II; nptIII, neomycin phosphotranspherase III; pnos, nos promoter; tnos, nos terminator; p35S, CaMV35S promoter. Restriction enzyme recognition sites in the multicloning site are indicated in the map.
