# Supplementary material for: Effects of the repression of GIGANTEA gene StGI.04 on the potato leaf transcriptome and the anthocyanin content of tuber skin
Source: BMC Plant Biol. 2022 May 20;22:249. doi: 10.1186/s12870-022-03636-3 (PMC9121593; doi:10.1186/s12870-022-03636-3)
Supplement: Supplementary file 2 — Additional file 2: Table S1. RNA-seq quality statistics. Table S2. Primer sequences. [file 12870_2022_3636_MOESM2_ESM.docx]

**Table S1**

**RNA-seq quality statistics**

| **Sample** | **Raw reads** | **Clean reads** | **Clean read %** | **Error rate** | ***Total mapped reads** | **Uniquely mapped reads** | **Total mapping rate %** | **Uniquely mapping rate %** |
| --- | --- | --- | --- | --- | --- | --- | --- | --- |
| **aGI52_1** | 44606327 | 43508838 | 97.5 | 0.03 | 78637147 | 75659748 | 90.4 | 87.0 |
| **aGI52_2** | 44261858 | 43308470 | 97.8 | 0.02 | 78614280 | 75647716 | 90.8 | 87.3 |
| **aGI52_3** | 45071778 | 44379759 | 8.5 | 0.03 | 80000956 | 77146601 | 90.1 | 86.9 |
| **DES_1** | 43461307 | 42406507 | 97.6 | 0.03 | 75373931 | 72617290 | 88.9 | 85.6 |
| **DES_2** | 44330084 | 43104447 | 97.2 | 0.02 | 78331213 | 75360702 | 90.9 | 87.4 |
| **DES_3** | 43702308 | 42121399 | 96.4 | 0.03 | 76200451 | 73548767 | 90.5 | 87.3 |

*Mapping was carried out to the potato reference genome *S. tuberosum* group Phureja DM 1-3 v6.1.

**Table S2**

**Primer sequences**

| **Name** | **Forward sequence (5’-3’)** | **Reverse sequence (5’-3’)** |
| --- | --- | --- |
| GI250 | *GAATTCABBATTGGAAGCACACCTAA | GGTACCGGCCAAATCTGAAGCATCTAA |
| GI.04spec | GTACGTGCACTCAGCATATCA | GCAGGACCATGGATACCATTT |
| GI.12spec | TGGCTTCTTCAAGCACAAGGT | GCGGTAATTTGATCCTTCCGC |
| ACTIN | TGGACTCTGGTGATGGTGTG | GGTTTCAAGTTCCTGTCTGT |
| EF1α | GACAAGCGTGTTATTGAGAGG | CACAGTGCAGTAGTACTTAGTG |
| SD1 | AAGGTTCCTTCAGGCGATGG | GTGCACTGTCTACTCTGTCCC |
| ERF1B | CGAAAACGTCCATGGGGAAA | ACATGTTGAAGGACCACGCA |
| LRR9 | GATCGTGGTTGGAATTGCCG | TCCGCGACAATTAGCCATGT |
| RAP2-7 | AGAAGCTGCAAGGGCGTAT | TTCCTTCGGAGAAGAAGTCGC |
| DTX48 | CCAACCCCATCTGAGGCTTT | AGAGACCCTCCTGCTAGCTC |
| CRF2 | TGGCCGTCAAAGAGTGAGAA | CTTCCGCCTTTTTGGTGGTG |
| NFYA1 | TTATGCCTCCAAGCATGGGT | ATGCAATTGAGGAGGGTGCC |
| HSP83 | TGGAAGCTCTACAGGCTGGG | GCCCAATTGCTCCTCATCAAC |

*The restriction enzyme recognition sites *Eco*RI and *Kpn*I are underlined.
